# Time-Course of Metabolic and Proteomic Responses to Different Nitrate/Ammonium Availabilities in Roots and Leaves of Maize

**DOI:** 10.3390/ijms19082202

**Published:** 2018-07-27

**Authors:** Bhakti Prinsi, Luca Espen

**Affiliations:** Department of Agricultural and Environmental Sciences—Production, Landscape, Agroenergy (DiSAA), Università degli Studi di Milano, Via Celoria 2, 20133 Milano, Italy; luca.espen@unimi.it

**Keywords:** ammonium, co-provision, maize, nitrate, plant nutrition, proteomics

## Abstract

The availability of nitrate and ammonium significantly affects plant growth. Co-provision of both nutrients is generally the best nutritional condition, due to metabolic interactions not yet fully elucidated. In this study, maize grown in hydroponics was exposed to different nitrogen (N) availabilities, consisting of nitrate, ammonium and co-provision. Roots and leaves were analyzed after 6, 30, and 54 h by biochemical evaluations and proteomics. The ammonium-fed plants showed the lowest biomass accumulation and the lowest ratio of inorganic to organic N content, suggesting a metabolic need to assimilate ammonium that was not evident in plants grown in co-provision. The N sources differently affected the root proteome, inducing changes in abundance of proteins involved in N and carbon (C) metabolisms, cell water homeostasis, and cell wall metabolism. Notable among these changes was that some root enzymes, such as asparagine synthetase, phosphoenolpyruvate (PEP) carboxylase, and formate dehydrogenase showed a relevant upsurge only under the sole ammonium nutrition. However, the leaf proteome appeared mainly influenced by total N availability, showing changes in the abundance of several proteins involved in photosynthesis and in energy metabolism. Overall, the study provides novel information about the biochemical determinants involved in plant adaptation to different N mineral forms.

## 1. Introduction

Nitrogen (N) is the mineral element required in the highest amount by plants: nitrate (NO_3_^−^) and ammonium (NH_4_^+^) represent the main inorganic N sources [1]. Since the proportion in which NO_3_^−^ and NH_4_^+^ are available in agricultural soils strongly affects crop productivity [2], a better understanding of plant biochemical responses to N sources could help to improve agricultural sustainability. Nitrate and NH_4_^+^ have different, and sometimes opposite, effects on plant development, growth rate, root architecture, and leaf expansion [3]. The use of NO_3_^−^ and NH_4_^+^ by plants is sustained by different mechanisms of acquisition, allocation, and assimilation [2,4,5]. After uptake by roots, NO_3_^−^ is firstly reduced by nitrate reductase (NR) and nitrite reductase (NiR) to generate NH_4_^+^, which, together with the quota derived from soil and metabolism, is assimilated into amino acids by the glutamine synthetase/glutamate synthase pathway (GS/GOGAT) [2]. The contribution of roots and leaves in N assimilation is influenced by several factors, but in both cases, the process involves several interactions with carbon (C) metabolism that allow the plant to sustain the requirements of C skeletons and of metabolic energy [6]. The use of NO_3_^−^ or NH_4_^+^ by plants is associated with different balancing among glycolysis, the oxidative pentose pathway, and the tricarboxylic acid (TCA) cycle [2,7]. Considering the theoretical metabolic costs [8] and the demand for reducing equivalents [9], the use of NH_4_^+^ by plants seems to be advantageous compared to that of NO_3_^−^, but this prediction does not often coincide with empirical observations [3]. An exclusive, or excessive, NH_4_^+^ nutrition could have adverse impacts on plants, including alterations in root metabolism, plant ionic imbalances, and foliar oxidative stress [10,11]. Plant responses also significantly depend on the relative proportion between the NH_4_^+^ and the NO_3_^−^ available in the soil. The co-provision of both NH_4_^+^ and NO_3_^−^ is generally considered the optimal N condition, in which the two nutrients reveal synergistic beneficial effects [12]. The synergy mainly arises from the reciprocal influences between NH_4_^+^ and NO_3_^−^ on their uptake, on root morphology, on the transport of N compounds from roots to shoots, and on plant C metabolism. Overall, these interactions improve the capabilities of the plants for N acquisition and assimilation [13]. Large-scale approaches have turned out to be very useful to investigate the complexity of N nutrition in plants, as proven by many transcriptomic studies conducted in *Arabidopsis thaliana* [11,14]. However, several aspects have yet to be fully elucidated, such as the interactions and communications between NO_3_^−^ and NH_4_^+^ and between roots and leaves.

Maize (*Zea mays* L.) is a crop of worldwide economic relevance, and is characterized by C4 metabolism and a very high demand for N inputs in agricultural systems [15]. Some large-scale studies have been devoted to investigating NO_3_^−^ metabolism in maize, providing new evidence that this anion acts as a signal influencing its uptake and assimilation both at transcriptional and protein levels [16,17,18]. Moreover, these studies have been useful in revealing new aspects regarding the interlinks between C and N metabolism in plants [19,20]. In particular, comparative proteomics shows that NO_3_^−^ availability evokes different responses in roots and leaves, highlighting the importance of analyzing both organs [20]. To our knowledge, similar approaches have not yet been applied in maize to study the responses to NH_4_^+^, either as sole N nutrient or in combination with NO_3_^−^. 

In this study, maize plants were exposed to NO_3_^−^, to NH_4_^+^, or to co-provision and analyzed over a period of three days, in order to appreciate plant metabolic and biochemical differences. The evaluation of plant growth and nutritional status in roots and leaves was combined with a comparative proteomic approach. This investigation showed that NO_3_^−^ and NH_4_^+^ had different effects on plant growth and that the availability of NO_3_^−^ in co-provision affected the accumulation and assimilation of NH_4_^+^ in roots and leaves. Interestingly, the root proteome was more specifically influenced by the N source, while leaf profiles were mainly affected by the total N availability. Moreover, some proteomic changes were specifically induced by NH_4_^+^ as sole nutrient and absent in co-provision or with NO_3_^−^ nutrition. Taken together, the results suggest that NO_3_^−^ availability influenced the capability of the plants to manage the content of NH_4_^+^ in the cells, probably due also to its action as an osmolyte. Moreover, the study contributes to a better understanding of plant N metabolism, providing novel information about molecular mechanisms involved in plant adaptation to different N sources.

## 2. Results and Discussion

This study was devised to investigate biochemical responses specific to NO_3_^−^ or NH_4_^−^ availability, as well as the interactions between the two nutrients in co-provision, in maize plants during early vegetative growth. Seedlings were grown in a hydroponic system with low N availability (1 mM NO_3_^−^, 125 µM NH_4_^+^) for a total of nine days until the expansion of the second leaf, and then exposed to one of three N treatments: 5 mM NO_3_^−^ (n); 5 mM NH_4_^+^ (a); 2.5 mM NO_3_^−^ + 2.5 mM NH_4_^+^ (na). This experimental design was chosen in order to expose maize seedlings to the same availability of total N, while changing the proportion between NO_3_^−^ and NH_4_^+^. Moreover, since maize is generally fertilized by a single application at sowing [21], this growth stage corresponds to a period in which maize plants are exposed to high levels of N and in which they often show the highest susceptibility to an excess of NH_4_^+^ [22]. Plants were analyzed for a period of three days (t0, 6 h, 30 h, and 54 h) to appreciate both early biochemical responses and metabolic acclimations in roots and leaves. After the evaluation of plant growth, nutritional parameters were analyzed in combination with proteomic changes in both organs.

### 2.1. Plant Growth and Metabolic Status of Roots and Leaves

**Figure 1:**
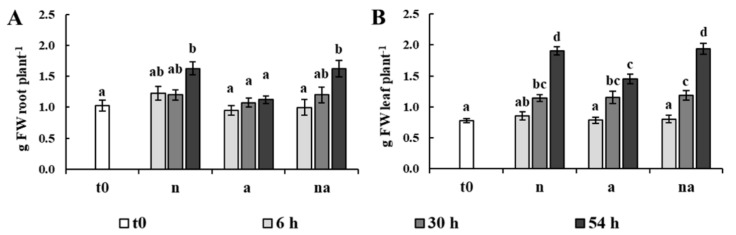
Plant growth evaluated as the fresh biomass of roots (**A**) and leaves (**B**) per plant (g FW plant*^−^*^1^). Maize plants were collected at t0 (white bar) or after 6 h (light grey bars), 30 h (grey bars) and 54 h (dark grey bars) of growth in the presence of 5 mM NO_3_*^−^* (n), 5 mM NH_4_^+^ (a) and of 2.5 mM NO_3_*^−^* + 2.5 mM NH_4_^+^ (na). Values are the mean ± standard error (SE) (*n* = 8). The statistical significance was assessed by analysis of variance (ANOVA) test (*p* < 0.05, Tukey post hoc method).

The estimation of plant growth, measured as the biomass of roots and leaves during the different nutritional treatments, revealed that the two organs grew with a different dynamic (Figure 1).

In plants exposed to NO_3_^−^ and to co-provision (n, na), root growth was appreciable at the third day, resulting in a biomass increment of about 60% compared with the plants at t0. However, leaf growth was already evident after 30 h in all conditions, leading to a doubling of the biomass at 54 h (Figure 1). This behavior is in agreement with the fact that an increase in N supply promotes an increase in biomass shoot/root ratio in several plant species [3,23]. Although this observation was similar in all conditions, plants supplied only with NH_4_^+^ (a) were characterized by a much slower increase in the fresh weight of the roots and showed the lowest leaf growth (Figure 1). These results confirm previous studies reporting that NH_4_^+^-fed maize plants accumulate less biomass than those fed with NO_3_^−^ [24]. Plants grown in co-provision (na) showed a biomass accumulation very similar to that of the NO_3_^−^-fed plants (n, Figure 1), suggesting that the lower level of NH_4_^+^ in the growth medium and/or the presence of NO_3_^−^ led to a reduction of the negative effects caused by a sole NH_4_^+^ nutrition.

**Figure 2:**
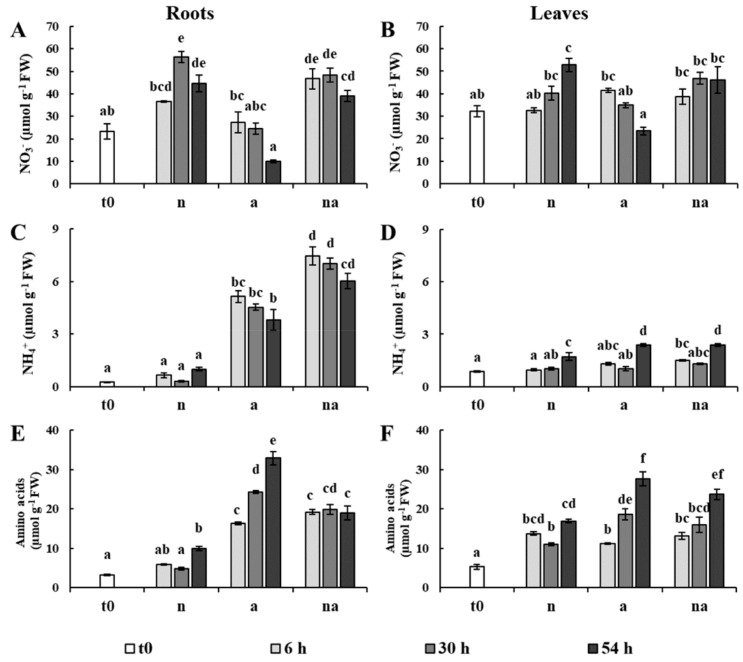
Content of NO_3_*^−^*, NH_4_^+^ and amino acids in roots and leaves. The graphs report the content of NO_3_*^−^* in roots (**A**) and in leaves (**B**); the content of NH_4_^+^ in roots (**C**) and in leaves (**D**); the content of amino acids in roots (**E**) and in leaves (**F**). Maize plants were collected at t0 (white bar) or after 6 h (light grey bars), 30 h (grey bars), and 54 h (dark grey bars) of growth in presence of 5 mM NO_3_*^−^* (n), 5 mM NH_4_^+^ (a), and 2.5 mM NO_3_*^−^* + 2.5 mM NH_4_^+^ (na). Values are the mean ± SE (*n* = 3). The statistical significance was assessed by ANOVA test (*p* < 0.05, Tukey post hoc method).

The levels of NO_3_^−^, NH_4_^+^, and amino acids were determined in roots and leaves (Figure 2).

In the (n) condition, the content of NO_3_^−^ reached the highest level at 30 h in roots and at 54 h in leaves, which in comparison to the t0 plants corresponded to an increment of about 140% and 45%, respectively (Figure 2A,B). The plants grown in co-provision (na) showed a similar accumulation of NO_3_^−^ in both organs, even though the availability of the anion in the growth medium was only half (Figure 2A,B). This observation indicates that the accumulation of the anion was not proportional to the external availability, but it was probably regulated by the requirements of the plants. Moreover, the copresence of NH_4_^+^ did not seem to modify this process. These results seem to be inconsistent with the observation that NO_3_^−^ uptake is reduced in presence of NH_4_^+^. However, in barley (Hordeum vulgare L.) and in Arabidopsis this effect was ascribed to an inhibition of the inducible high affinity transport systems [25,26], and it is therefore conceivable that it did not influence the accumulation of NO_3_^−^ in plants exposed to high N input (>1 mM) for several hours. At the same time, the (a) plants showed a gradual decline in NO_3_^−^ levels in roots and leaves (Figure 2A,B), probably because the plants continued to use the NO_3_^−^ reserves in the presence of NH_4_^+^.

In (n) plants, the content of NH_4_^+^ did not change in roots, while it showed some fluctuations in leaves (Figure 2C,D). However, in plants exposed to NH_4_^+^ and to co-provision (a, na) the content of the cation greatly increased, especially in roots, but it never reached levels associated with NH_4_^+^ toxicity in maize [22,27]. In these conditions (a, na), in roots the NH_4_^+^ content already surged up at 6 h, while an increase of foliar NH_4_^+^ was evident only after 54 h (Figure 2C,D).

In this context, it is important to note that in (a) plants, 54 h of treatment led to both the highest accumulation of NH_4_^+^ and to an almost total depletion of NO_3_^−^ (Figure 2). However, the roots of the plants in co-provision (na) were characterized by an NH_4_^+^ content much higher than those of the (a) plants, even though the external availability was only half (Figure 2C).

The amino acid levels rose in both organs in all the nutritional conditions. This increase was higher in plants exposed to NH_4_^+^ and to co-provision (a, na, Figure 2E,F), in agreement with the observation that, in maize, ammonium-fed plants show higher contents of amino-N compounds than nitrate-fed ones [24]. Interestingly, in roots the accumulation of amino acids was not proportional to the content of NH_4_^+^. Indeed, the (a) roots were characterized by an upsurge of amino acid levels at 54 h, which was not evident in (na) plants (Figure 2E). These results suggest that the availability of NO_3_^−^ could have exerted positive effects on the storage capacity of NH_4_^+^ of the plants.

To investigate this hypothesis, we calculated the total content of N in plants as well as the ratio between the inorganic N and organic N in roots and leaves (Table 1). The total N content in plants at 54 h reached similar values in all the nutritional treatments. However, plants showed very different partitioning between inorganic and organic forms of N. The (a) plants were characterized by a particular decrease in this ratio, due to the highest increase in amino acid and protein levels. On the contrary, plants in co-provision showed values more similar to (n) plants, although they had the highest NH_4_^+^ content in roots (Figure 2). These results indicate that in co-provision the presence of NO_3_^−^ promotes a major capability of storage of NH_4_^+^ in the roots, and therefore it could contribute to alleviating metabolic stress by reducing the need to assimilate the cation into amino acids.

**Figure 3:**
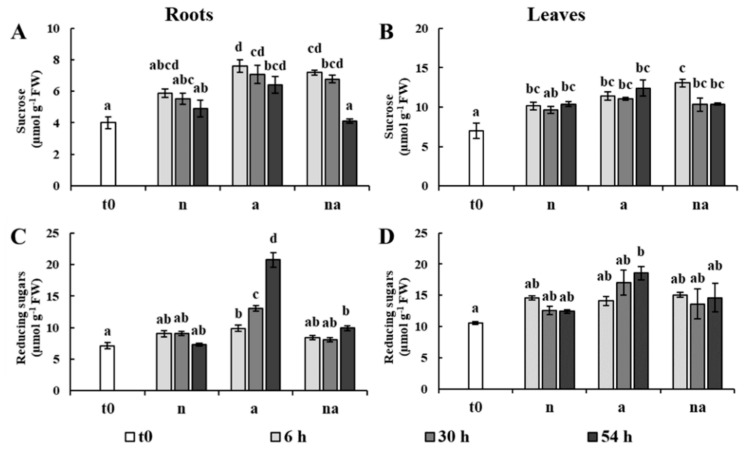
Contents of sucrose (**A**,**B**) and reducing sugars (**C**,**D**) in roots (**A**,**C**) and leaves (**B**,**D**). Maize plants were collected at t0 (white bar) or after 6 h (light grey bars), 30 h (grey bars), or 54 h (dark grey bars) of growth in presence of 5 mM NO_3_^−^ (n), 5 mM NH_4_^+^ (a) and 2.5 mM NO_3_^−^ + 2.5 mM NH_4_^+^ (na). Values are the mean ± SE (*n* = 3). The statistical significance was assessed by ANOVA test (*p* < 0.05, Tukey post hoc method).

The evaluation of the contents of sucrose and reducing sugars showed that, after a little increase at 6 h, the levels were only slightly affected by the nutritional treatments, except for a remarkable doubling in the content of reducing sugars in the roots of the (a) plants, not found in co-provision (Figure 3). Although this had already been observed in another maize genotype [27], further studies are needed to clarify the metabolic meaning of this accumulation. However, this result weakens the possibility that the lack of root growth of the (a) plants was due to an insufficient allocation of photoassimilates, as previously proposed in other plant species [2,10].

### 2.2. Comparative Proteomic Analyses in Roots and Leaves of Maize during Exposure to Different N Sources

The analysis of the proteomic changes in roots and leaves of maize plants exposed to different N sources (n, a and na) was done by comparing the total proteome of each organ among all the conditions (three nutritional treatments for three timings). The comparison was performed by means of gel liquid chromatography-mass spectrometry (GeLC-MS/MS): proteins are purified by sodium dodecyl sulphate-polyacrylamide gel electrophoresis (SDS-PAGE), in-gel digested, and then identified and quantified by mass spectrometry [28]. This approach allowed us to analyze the abundance of 336 and 246 proteins in roots and leaves, respectively, with high reliability in identification and a good degree of comparability among samples and conditions (Table 2, Supplementary Data 1).

The proteins that showed changes in abundance of at least two-fold in at least two conditions were further selected by means of the two-way ANOVA to find the main source of variation (time, N sources), and by means of the Tukey-test (p < 0.05) to evaluate the differences among conditions. The selected proteins were named Differentially abundant Proteins (DPs). The DPs identification in roots and leaves, their changes and statistics, are reported in Table 3 and Table 4, respectively.

**Figure 4:**
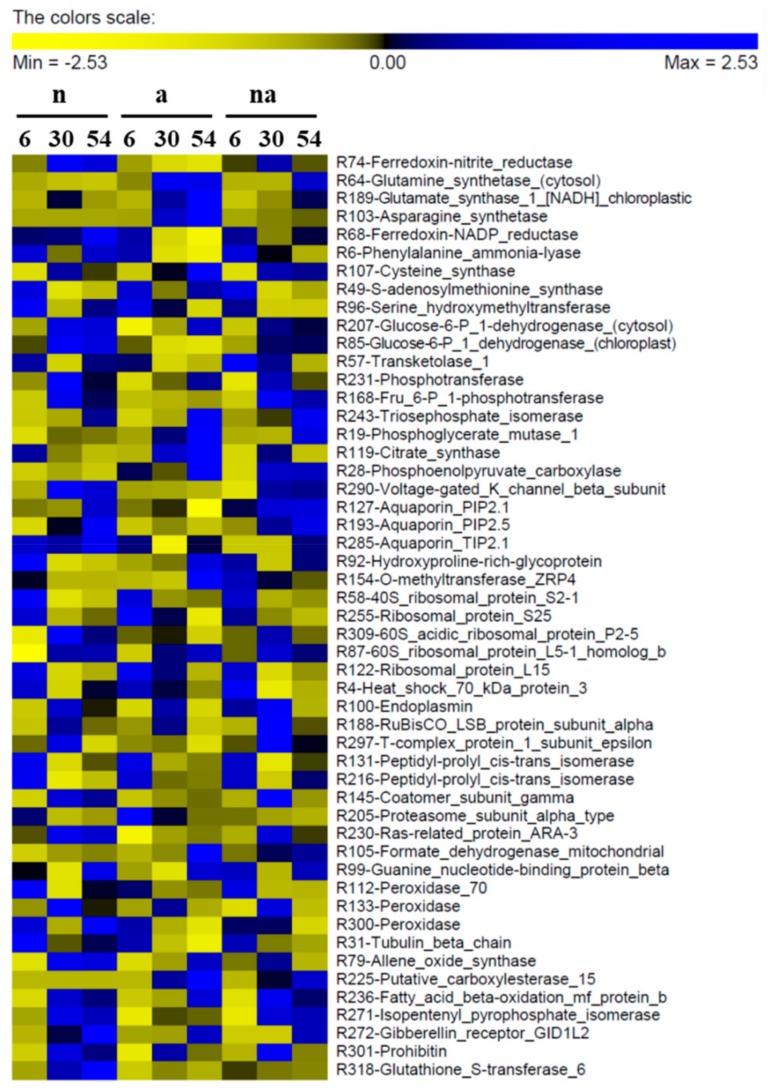
Abundance of the differentially accumulated proteins in maize roots. Maize plants were exposed for 6, 30, and 54 h to the presence of 5 mM NO_3_^−^ (n), 5 mM NH_4_^+^ (a), and 2.5 mM NO_3_^−^ + 2.5 mM NH_4_^+^ (na). The image was obtained by means of the PermutMatrix graphical interface after Z-score normalization of the averages of protein Spectrum Intensity % (%SI, *n* = 3). Each colored cell represents the average of the %SI according to the color scale.

**Figure 5:**
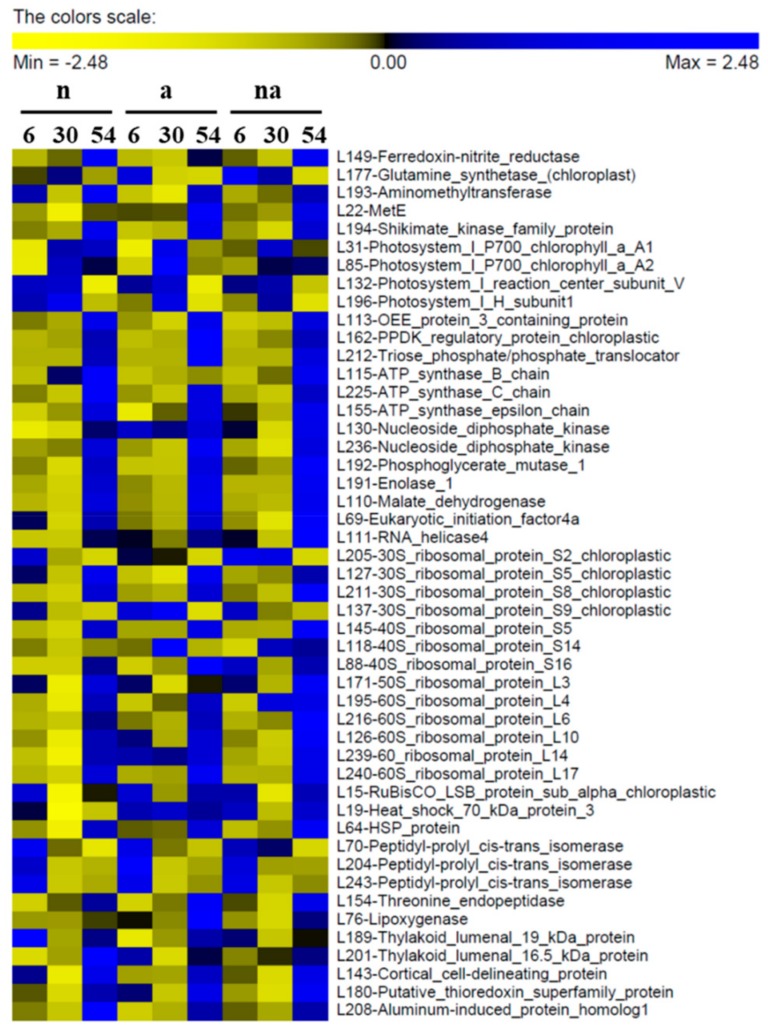
Abundance of the differentially accumulated proteins in maize leaves. Maize plants were exposed for 6, 30, and 54 h to the presence of 5 mM NO_3_^−^ (n), 5 mM NH_4_^+^ (a), and 2.5 mM NO_3_^−^ + 2.5 mM NH_4_^+^ (na). The image was obtained by means of the PermutMatrix graphical interface after Z-score normalization of the averages of protein Spectrum Intensity % (%SI, *n* = 3). Each colored cell represents the average of the %SI according to the color scale.

The cluster representations of the DP abundances in roots and leaves are shown in Figure 4 and Figure 5 (for detailed bar graphs reporting protein levels see Supplementary Data 2).

The DPs accounted for 15% and 19% of the quantified proteins in roots and leaves, respectively (Table 2), showing that the two organs were affected by the treatments to a similar extent. This result is in agreement with our previous two-dimensional gel electrophoresis (2-DE) study, showing comparable effects on root and leaf proteome in maize plants exposed to NO_3_^−^ [20]. In contrast, a microarray study in Arabidopsis indicated that the responses to NO_3_^−^ were much more ample in roots than in shoots [29], but this discrepancy probably derives from different approaches and/or nutritional treatments. 

**Figure 6:**
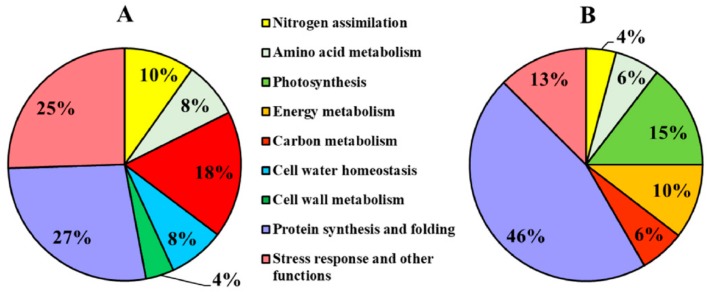
Functional distribution of the differentially accumulated proteins in maize plants. The proteins differentially accumulated were grouped in classes according to literature and GeneBank. (**A**) Proteins differentially accumulated in roots; (**B**) proteins differentially accumulated in leaves. The functional distribution indicates the percentage of each class as compared to the total number of proteins differentially accumulated.

The functional classification of the DPs recognized nine main classes, partially overlapping in roots and leaves (Figure 6). “Protein synthesis and folding” was the main category in both organs, indicating a general reprogramming of plant functionalities. Leaves were also characterized by changes in DPs involved in photosynthesis (15%) and energy metabolism (10%), while in roots the DPs related to C metabolism (mainly catabolic processes) were predominant (18%). Moreover, the root proteomic profile was characterized by changes in proteins involved in cell water homeostasis (8%) and cell wall (4%).

**Figure 7:**
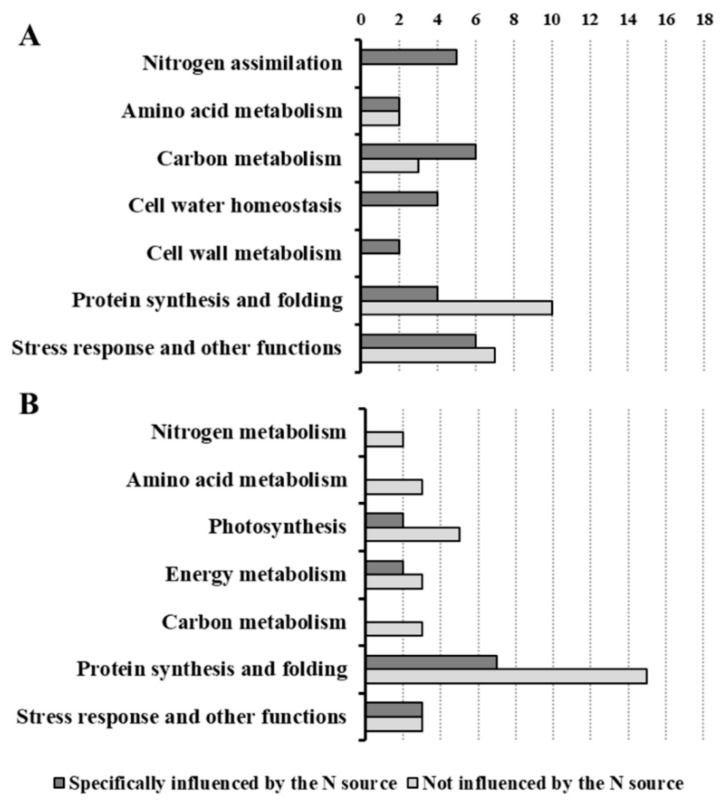
Classification of the differentially accumulated proteins according to the main source of variation in roots (**A**) and in leaves (**B**). The proteins differentially accumulated, sorted in functional classes, are categorized in two groups: proteins whose changes were specifically related to the N source (dark grey bars) and proteins whose changes were not related to N sources, but to other factors, such as time and the total N availability (light grey bars).

The classification of DPs according to the main source of variation (Figure 7, Table 3 and Table 4, Supplementary Data 1) discriminated between the DPs specifically affected by the N source and the DPs that were not influenced by this factor (changes in which were related to time and/or to total N availability). In roots, most of the DPs were specifically affected by the N source (Figure 7A), highlighting that NO_3_^−^ and NH_4_^+^ led to distinct effects. This result is in agreement with a study showing that in Arabidopsis plants exposed for 1.5 h to NO_3_^−^ or NH_4_^+^ more than 40% of the transcriptomic changes in roots were nitrate- or ammonium-specific [30]. However, in the leaf proteome the DPs were more equally distributed between the two categories (Figure 7B), indicating that the leaf metabolism was less specifically affected by the kind of N source respect than the root one. Overall, this response was consistent with the physiological and biochemical data. Indeed, the treatments differently affected root growth but all of them sustained an increment in leaf biomass. Moreover, the nutritional treatments induced metabolic changes which were more different among conditions in roots than in leaves (Figure 1, Figure 2 and Figure 3). As highlighted by previous studies [20,22,27], this proteomic profiling confirmed the fundamental role of roots in plant adaptation to the kind of N source.

### 2.3. Proteomic Changes Involved in Nitrogen (N) Assimilation and Amino Acid Metabolism

The proteomic analysis showed that the nutritional treatments induced changes in the levels of Ferredoxin-Nitrite Reductase (Fd-NiR) in roots (R74, Figure 4) and in leaves (L149, Figure 5), with trends similar to the contents of NO_3_^−^ in the organ (Figure 2). This was particularly evident in (n) plants, in which Fd-NiR reached the maximum level after 30 h and 54 h in roots and leaves, in conjunction with the peak of NO_3_^−^ accumulation. Moreover, the Fd-NiR levels in (na) plants (Figure 4 and Figure 5) suggest that NO_3_^−^ reduction was sustained even in the presence of NH_4_^+^.

Considering that plastid Fd-NADP^+^ reductase (FNR, R68, Table 3) reduces the Fd-like electron carrier for NiR [31], it is of interest that FNR and Fd-NiR showed similar profiles in roots (Figure 4). These results confirmed that both enzymes are strictly coordinated and take part in the “root primary response to NO_3_^−^” [29,32].

On the contrary, the trends observed for two glutamine synthetases (GS) were dissimilar in roots and leaves, probably because the two enzymes were different isoforms with well-known specific roles. In leaves, the enzyme (L177, Table 4) belongs to the chloroplast GS2 type (92.8% of identity with GS2 P25462) which plays a pivotal role in NO_3_^−^ assimilation. Its decrease in the course of time (Figure 5) could be due to leaf age and/or to amino acid accumulation, as previously observed [33]. Instead, in roots GS was identified as the cytosolic GS1-1 isoform (R64, Table 3, 99.7% of identity with GS1-1 P38559) that surged up in plants exposed to NH_4_^+^ (a, na, Figure 4), confirming its role in NH_4_^+^ assimilation [27].

The concurrent increases of GS1, of the glutamate synthase 1 [NADH] chloroplastic (R189, Table 3) and of the amino acid levels in roots of (a) plants (Figure 2F and Figure 4) indicated a relevant induction of NH_4_^+^ assimilation. Interestingly, all these traits were lower in the roots of the (na) plants, even if the NH_4_^+^ content was higher (Figure 2C and Figure 4), confirming that the copresence of NO_3_^−^ could somehow reduce the need to quickly assimilate the NH_4_^+^ ions (see below).

This hypothesis was further supported by the profile of the asparagine synthetase (AS), the enzyme that catalyzes the ATP-dependent synthesis of asparagine by the transfer of the amino group from glutamine to aspartate. In several plant species, some members of the AS gene family are upregulated by increases in the levels of amino acids and/or of internal NH_4_^+^, suggesting that AS could contribute to its assimilation during nutritional stress conditions [34]. In our study, one AS (R103) was greatly induced in the roots of the NH_4_^+^-supplied plants (a), while it was almost undetectable in plants exposed to nitrate or to co-provision (n, na, Figure 4), confirming that the presence of NO_3_^−^ could reduce the stress induced by NH_4_^+^ accumulation in roots.

In this context, it is important to note that it was proposed that glutamate dehydrogenase (GDH) could contribute to the assimilative process when plants are exposed to an excessive NH_4_^+^ nutrition [2]. Our proteomic analysis revealed that in roots GDH did not change in abundance during any treatment (R104, Supplementary Table S1). This observation is in agreement with the fact that the NH_4_^+^ content in roots never reached levels associated with toxicity in maize [22,27], but it does not exclude the idea that the enzyme could have relevant roles at higher NH_4_^+^ inputs or during longer exposures.

On the whole, the proteomic analysis provided evidence that co-provision could also have positive effects on plants’ growth because of the ability of NO_3_^−^ to change the balance between the quota of NH_4_^+^ drained by assimilation and the quota of NH_4_^+^ delivered to vacuole storage.

At the same time, the proteomic analysis revealed changes in the levels of enzymes involved in amino acid metabolism in roots and leaves, most of which were not specifically related to the N source (Figure 7). In leaves, enzymes involved in glycine (L193), methionine (L22), and aromatic amino acid (L194) metabolisms increased in abundance over time (Table 4, Figure 5), in agreement with a raising of the leaf anabolic processes after the N inputs. In roots, the members of this class were differently affected as regards both trends and sources of variation. The levels of phenylalanine ammonia-lyase (PAL) decreased in plants exposed to NH_4_^+^, both during (a) and (na) treatment (R6, Table 3, Figure 4). These data suggest that, similarly to NO_3_^−^ [20,35], the NH_4_^+^ contents could also exert negative feedback at the enzyme level, probably to reduce additional release of the cation via PAL activity.

### 2.4. Changes in Proteins Involved in Photosynthesis, Energy, and Carbon Metabolism

The leaf proteome was characterized by DPs involved in photosynthesis and energy metabolism, many of which were mainly influenced by time and/or by the total N availability (Figure 6 and Figure 7). The increases in the Photosystem I P700 chlorophyll a apoproteins A1 and A2 (L31, L85, Table 4, Figure 5), which are the large subunits PsaA and PsaB that form the core of the Photosystem I complex (PSI) [36], support the hypothesis that a general increase of PSI functionality occurred to sustain N assimilation. 

On the other hand, in leaves in all conditions (n, a, and na) at 54 h, several events occurred at once, along with the increase in NH_4_^+^ and amino acid content (Figure 2). Firstly, it was possible to observe a decrease in abundance of the PSI reaction center subunit V (L132, also known as PsaG) and of the PSI H subunit1 (L196), which are involved in the interaction of the PSI with the light harvesting complexes I (LCHI) and LCHII, respectively [36]. In addition, it was possible to observe an increase of the oxygen evolving enhancer (OEE) protein 3 (L113), which belongs to the photosystem subunit Q (PsbQ) family involved in stabilizing the PSII-LCHII complex [37]. These results support the hypothesis that, at 54 h, the leaves went through a modulation of the energy balance between the two Photosystems (i.e., a transition from State II to State I) that promoted PSII functionality and linear electron flow. This condition corresponds to an optimization of the photosynthetic machinery to generate both adenosine triphosphate (ATP) and reduced form of nicotinamide adenine dinucleotide phosphate (NADPH) required by the Calvin Cycle [38]. It coincided with an increase of three subunits of chloroplast ATP synthase (L115, L225, and L155) and of the pyruvate phosphate dikinase regulatory protein (L162), which regulates CO_2_ fixation in C_4_ plants [39]. Finally, the concomitant increases of the triose phosphate/phosphate translocator (L212), which mediates the export of fixed carbon from chloroplast to cytosol [40], of two nucleoside diphosphate kinases (L130 and L236), cytosolic enzymes involved in balancing between the pools of ATP and the nucleosides [41], as well as of two glycolytic enzymes (L192 and L191) and of the mitochondrial malate dehydrogenase (L110) suggested a general upsurge in energy production and in respiratory metabolism (Table 4, Figure 5). On the whole, considering that in plants the maintenance of low levels of NH_4_^+^ in tissues is one of the main strategies to avoid metabolic stresses [11], it is possible to propose that, at 54 h, the NH_4_^+^ accumulation in leaves led to an increment of the photosynthetic machinery and of C metabolism to sustain the synthesis of amino acids. In this regard, the high content of reducing sugars in the roots of the (a) plants at 54 h could indicate that a massive allocation of photoassimilates in this organ occurred when NH_4_^+^ was provided as the sole N nutrient (Figure 3C). This response, curiously absent in co-provision, could be one of the causes contributing to the slower leaf growth (Figure 1B).

In roots, C metabolism was instead more specifically affected by the kind of N source (Table 3, Figure 7). Seeing that the oxidative pentose pathway is induced by NO_3_^−^, as indicated in maize root plastids [42] and by transcriptomics in Arabidopsis roots [30], it is important to analyze the changes of two isoforms of glucose-6-phosphate 1-dehydrogenase (G6PDH, Table 3). The first one, the R207, which is probably a cytosolic form (80% of identity with P37830, a cytoplasmic isoform of S. tuberosum), increased with time. However, the second one, the R85, which belongs to the plastid protein cluster, showed a higher level in (n) and (na) plants, and it almost disappeared in (a) roots after 54 h (Table 3, Figure 4). These results are in agreement with the fact that in barley (H. vulgare L.) the cytosolic G6PDH activity seems to be correlated with general growth processes, while the plastidic one is induced to a higher extent by NO_3_^−^ than by NH_4_^+^ [43]. 

Similarly, some root glycolytic enzymes were differently affected by the nutritional treatments (Table 3). The pyrophosphate-fructose 6-phosphate 1-phosphotransferase (R168, also known as phosphofructokinase) was induced by NO_3_^−^ in (n) and (na) roots (Figure 4). Although some studies exclude a key role for this enzyme in the control of glycolysis [44], in our opinion, this response deserves further investigation. Moreover, since changes in the phosphoglycerate mutase levels seem to have dramatic effects on metabolism [44], the increases in abundance of this enzyme (R19), together with citrate synthase (R119) and PEP carboxylase (R28) suggested an upsurge of respiratory metabolism in roots of the (a) plants at 54 h (Figure 4). With this in view, these results confirm that in roots of NH_4_^+^-fed plants the PEP carboxylase could play an important anaplerotic role for TCA replenishment, as previously proposed by several authors [2,10,45]. Taken together, these results provide new evidence that NO_3_^−^ and NH_4_^+^ have different effects on C metabolism in roots, according to different requirements for reducing power and C skeletons.

Overall, this proteomic study highlights the different metabolic roles for roots and leaves and it allows us to propose novel molecular determinants involved in the adaptation of plants to different N sources.

### 2.5. Root Proteomic Changes Involved in Cell Water Homeostasis and Cell Wall Metabolism

The root proteomic profile was characterized by DPs involved in cell water homeostasis and cell wall metabolism that were all specifically affected by the N source (Table 3, Figure 6A and Figure 7A). Considering the interactions between K and N nutrition on ion uptake, transport, and assimilation in plants [46], it is appropriate to highlight that the β subunit of a V-gated K^+^ channel (R290, Table 3) was specifically induced in (n) plants, where it reached its highest abundance after 30 h. Although to lesser extent, this effect was also appreciable in roots of the (na) plants but it was absent in the NH_4_^+^-fed plants (Figure 4). This observation could be associated with the fact that, at high external concentrations, the acquisition rates of NO_3_^−^ and K^+^ are often positively correlated ([46] and references therein), a relation that in barley roots was recently attributed to the stimulative effect of NO_3_^−^ on the K^+^ low-affinity influx system [47]. Whether this effect derives from an improvement in cell electrical balance and/or from a regulative molecular mechanism is as yet an unresolved question, which deserves future physiological and molecular studies. 

The N sources also differently affected the accumulation of some aquaporins located at both plasma membrane (PIP) and tonoplast (TIP) (Table 3). In particular, PIP2.1 (R127) maintained the highest levels in (n) and (na) roots but decreased by half at 54 h in (a) plants, while PIP2-5 (R193) was specifically accumulated in roots exposed to NO_3_^−^ (n and na, Figure 4). Several authors have proposed that the increment in root hydraulic conductivity during NO_3_^−^ exposure involves changes in PIP functionality [48]). However, a study conducted in maize indicated that NO_3_^−^ induces an increase in root hydraulic conductivity, but it does not correlate with any changes in the expression of the aquaporin genes, at least within 4 h of treatment [49]. It seems likely that the discrepancy between that study and our proteomic profiles derives from different exposure timing. For instance, it is possible that 54 h NO_3_^−^ accumulation in root tissues (Figure 2A) could have had effects on aquaporin abundances. Moreover, TIP2.1 (R285) is very similar to the aquaporin AtTIP2-3 (77% identity with Q9FGL2) which in Arabidopsis is involved in NH_3_ transport into the vacuole [50]. This protein was more abundant in the roots of (n) plants (Figure 4), suggesting that high NH_4_^+^ external inputs could exert inhibitory effects on the channel. 

Although future studies are needed for a conclusive verification, these observations allow us to propose that the presence of NO_3_^−^ might promote a higher accumulation of K^+^ channels and aquaporins in roots, maybe acting as an osmolyte. Considering that both protein families are involved in the NH_4_^+/^NH_3_ transport [11], it is possible to conceive some relationship between their induction by NO_3_^−^ and the highest NH_4_^+^ content in roots in co-provision (Figure 2D).

Furthermore, several authors have proposed links among aquaporin expression, cell water potential, and cell expansion in growing tissues [48]. Starting from these considerations, it is interesting to correlate the changes in aquaporins with the changes of the DPs involved in cell wall metabolism in roots. The proteomic analysis revealed that a hydroxyproline-rich glycoprotein dramatically decreased in abundance after 54 h of (n) nutrition (R92), while O-methyltransferase ZRP4 (R154) was specifically accumulated after 54 h of (a) exposure (Table 3, Figure 4). Since in maize roots these proteins are associated with the lignification and suberization of the secondary cell wall [51,52], these results could suggest that NO_3_^−^, in contrast with NH_4_^+^, induced changes sustaining root growth and development. This hypothesis is also consistent with the differences in root growth in (n) and (a) plants (Figure 1A). This topic deserves future investigation, which could also contribute to elucidating the molecular mechanisms underlying the differences in root morphology between NO_3_^−^- and NH_4_^+^-fed plants.

### 2.6. Proteomic Changes Related to Protein Synthesis and Folding

The “protein synthesis and folding” was the major functional class both in roots and leaves, accounting for 27% and 46% of the DPs, respectively (Figure 6). In both cases, most of them were not specifically affected by the N source (Figure 7), probably because the modulation of protein synthesis was mainly associated with plant growth after exposure to high N availabilities. This class encompasses many kinds of proteins, among which are an initiation factor (L69), three heat shock proteins (R4, L19, and L64), and an endopeptidase (L154) (Table 3 and Table 4). 

Ribosomal proteins accounted for 36% and 59% of the category in roots and leaves, respectively, and, in particular, in leaves they were identified as both chloroplastic (30%) and cytosolic (70%) members (Table 3 and Table 4). In general, the trends observed were very specific for each ribosomal DPs (Figure 4 and Figure 5), probably due to a modulation of the ribosome composition. This conclusion is in agreement with a previous study showing that the replenishment of NO_3_^−^ in N-starved Arabidopsis seedlings induces changes in the expression of more than 100 genes encoding ribosomal proteins [53]. However, it is very difficult to draw conclusions about the biochemical meanings of these changes because of the complexity that characterizes the plant ribosome. Plant ribosomes are composed of a large number of heterogeneous proteins grouped into many families, currently numbering 80 in the Arabidopsis genome [54], encoded by several gene paralogs, with specific developmental roles, for which transcriptional regulation is still unclear [55,56]. To unravel these aspects is far beyond the aims of this work, but we believe that the information provided by this proteomic profiling can be useful for future studies dealing with this fundamental issue of plant biology.

Several peptidyl-prolyl cis-trans isomerases (PPIases) of the cyclophilin-type (Cyp), namely, two in the roots (R131 and R216, Table 3) and three in leaves (L70, L204, and L243, Table 4) decreased in abundance in all the nutritional treatments over time (Figure 4 and Figure 5). The PPIases catalyse the cis-trans isomerization of prolyl bonds in polypeptide chains avoiding the accumulation of misfolded proteins, both during de novo synthesis and in restructuring of mature polypeptides [57]. In plants, one of the first Cyp was discovered in maize and its function was related to responses to abiotic stresses [58]. Moreover, in humans, Cyp18 seems to be involved in the elimination of damaged proteins accumulated under oxidative stress [59]. Since oxidative stress and protein synthesis alterations are often associated with nutrient deficiency, it is possible that the decline of these proteins was due to the exposure of the plants to high N inputs. This result supports the hypothesis that the Cyp proteins could contribute to plant defense responses during nutritional shortages.

Summing up, this proteomic profiling provides new evidence that the modulation of protein synthesis is a crucial and multi-faceted element in plant adaptation to N availability, which requires coordination of several protein families.

### 2.7. Stress Responses and Other Functions

The DPs related to stress responses and other functions allow us to point out two interesting differences between (a) and (na) plants, which are therefore probably related to the presence of NO_3_^−^ in co-provision. In particular, after 54 h of treatment, (a) plants were characterized by a relevant upsurge in abundance of formate dehydrogenase (R105) in roots and of lipoxygenase (L76) in leaves (Table 3 and Table 4, Figure 4 and Figure 5). Both increases were not significant in (na) plants, indicating that these responses were reduced by NO_3_^−^ availability although the NH_4_^+^ content was higher in roots and similar in leaves (Figure 2). 

Formate dehydrogenase is a mitochondrial enzyme that catalyzes the NAD^+^ dependent oxidation of formate to CO_2_ [60]. In non-photosynthetic tissues, one of the most plausible routes for formate production is the catabolism of serine and glycine [61]. It is conceivable that this metabolic condition may have occurred in response to the high accumulation of amino acids in roots during NH_4_^+^ nutrition, a trait that was less pronounced in co-provision (Figure 2). Moreover, in plants the induction of formate dehydrogenase is often induced by several abiotic stress, such as darkness and anoxia. Recently it was related with aluminium toxicity, caused by a metal cation whose detoxification shows several similarities with plant responses to NH_4_^+^, such as the production of organic acids and the vacuolar sequestration of this cation [62]. Considering that one-carbon metabolism could have roles in sustaining amino acid biosynthesis in non-photosynthetic tissues, our results suggest that formate dehydrogenase could be participating in mechanisms of tolerance to NH_4_^+^ in maize plants.

Plant lipoxygenases (LOX) are involved in polyunsaturated fatty acid and membrane metabolisms, but it has also been proposed that these enzymes could be accumulated as vegetative storage proteins in leaves, as observed in soybean (Glycine max L.) in response to sink limitation [63]. In one of our previous proteomic studies in maize, we were able to establish that the LOX identified in this analysis (codified by ZmLOX10) was accumulated in the leaf in response to high NO_3_^−^ supply (i.e., 10 mM NO_3_^−^, 30 h) [20]. Although it is not possible to exclude an involvement in the protection of stress induced by NH_4_^+^, all together these results support the hypothesis that LOX accumulation in the leaf could be a way to store N, a new and intriguing role of LOX in cereal crops. 

In addition, three root peroxidases of the class III (Prxs) showed significant changes in abundance (R112, R133, and R300, Table 3, Figure 4). Since Prxs play several roles in plants [64], it is difficult to assign specific biochemical meaning to these results. However, the relations among Prxs, cell wall metabolism in roots and N nutritional status in plants seem to be worthy of further investigation. Similarly, it is interesting to note that the broad class of “other functions” includes several proteins localized in plant cell organelles, such as a peroxisomal fatty acid beta-oxidation multifunctional protein (R236), a mitochondrial prohibitin (R301), and two thylakoid luminal 19 kDa and 16.5 KDa proteins (L189 and L201) with unknown function (Table 3 and Table 4). This observation leads us to assume that, within the next few years, subcellular proteomics studies will be very useful to obtain novel information about the roles played by cell organelles in plant adaptation to total N availability as well as to different N sources.

## 3. Materials and Methods

### 3.1. Plant Material and Nutritional Treatments

Maize (*Zea mays* L.) seeds of the line PR33A46 (Pioneer Hi-Bred Italia^®^, Gadesco Pieve Delmona, CR, Italy) were germinated in the dark at 26 °C for 72 h. Seedlings were then grown by a hydroponic system in a growth chamber with a 16/8 h day/night regime, at 26/22 °C, constant relative humidity of 65%, and PPFD of 500 μmol·m^−2^·s^−1^. After 48 h of incubation in 4 mM CaSO_4_, the plants were transferred into a growth solution with low N input (1 mM KNO_3_, 2 mM K_2_SO_4_, 0.875 mM KH_2_PO_4_, 0.5 mM MgSO_4_, 0.4 mM CaSO_4_, 62.5 μM (NH_4_)_2_SO_4_, 60 μM Fe-EDTA, 25 μM KCl, 12.5 μM H_3_BO_3_, 1 μM MnSO_4_, 0.25 μM CuSO_4_, 0.25 μM ZnSO_4_, 0.25 μM Na_2_MoO_4_, pH = 6.1). After six days, at the beginning of the day (t0), the plants were transferred into new growth solutions in which N availability was changed according to the following treatments (abbreviated by letters in brackets): (i) 5 mM NO_3_^−^ (n); (ii) 5 mM NH_4_^+^ (a); (iii) 2.5 mM NO_3_^−^ + 2.5 mM NH_4_^+^ (na). All the solutions were balanced with K_2_SO_4_ and continuously aerated by electric pumps. The plants were sampled at t0 and after 6, 30, and 54 h of treatment. Roots were rinsed with water and blotted with paper towels. Roots and leaves were separately collected, weighed and immediately frozen in liquid N_2_. Each biological sample was composed of roots or leaves collected from four plants. Samples were stored at −80 °C. The significance of the changes in the biomass accumulation in roots and in leaves (*n* = 8) was assessed by the ANOVA test (*p* < 0.05, Tukey post hoc method).

### 3.2. Determination of the Contents of Nitrate, Ammonium, Amino Acids, Sucrose and Reducing Sugars

Nitrate was extracted from leaf and root samples as previously described [20], and measured according to Cataldo et al. [65].

The contents of NH_4_^+^ in roots and leaves were measured by the *o*-phthalaldehyde (OPA) method, as described by Coskun and coworkers [66]. Briefly, samples were powdered in liquid N_2_, homogenized in 5 volumes of ice-cold 10 mM formic acid (FA), and centrifuged at 14,000 *g* for 10 min at 4 °C. The supernatants were filtered by Millipore Millex HC cartridges (0.45 μm). An aliquot of the extract was added to 3 mL of OPA reagent (100 mM KH_2_PO_4_, 100 mM K_2_HPO_4_, 3.75 mM OPA, 2 mM 2-mercaptoethanol (2-ME), pH = 7). After incubation for 30 min in the dark, the sample absorbance was determined at 410 nm. 

Amino acids, reducing sugars and sucrose were extracted from leaf and root samples as previously described [20]. Amino acid concentration was measured by the ninhydrin method [67], while the contents of sucrose and reducing sugars were determined according to Nelson [68]. The total content of N in roots and leaves was calculated as the sum of the inorganic N (derived from the contents of NO_3_^−^ and NH_4_^+^) plus the organic N (derived from the contents of amino acids and proteins, applying the conversion factor of 6.25 that is generally used in corn [69]). All of the analyses were replicated on three independent biological samples (*n* = 3) and compared by the ANOVA test (*p* < 0.05, Tukey post hoc method).

### 3.3. Protein Extraction, Gel Electrophoresis and in-Gel Digestion

Frozen samples of roots or leaves were powdered in liquid N_2_ using a mortar and pestle and an aliquot of 200 mg was suspended in 3 volumes of extraction buffer (150 mM Tris-HCl pH 6.8, 10% (*w*/*v*) glycerol, 2% (*w*/*v*) sodium dodecyl sulfate (SDS), 2% (*v*/*v*) 2-ME, 0.1 mg·mL^−1^ Pefablock (Fluka)). Samples were vortexed for 10 min at room temperature and incubated for 30 min at 90 °C. After centrifugation at 14,000× *g* for 15 min, the collected supernatants were recentrifuged at 14,000× *g* for 5 min. The supernatants were stored at −80 °C until further use. Protein concentration was measured by 2-D Quant Kit (GE Healthcare, Milan, Italy).

Protein samples were colored with traces of bromophenol blue and aliquots of 15 µg were purified by partial 1D SDS-PAGE (1-dimensional SDS-polyacrylamide gel electrophoresis) conducted on 16% (*w*/*v*) polyacrylamide gel [70], monitoring by the Full-Range Rainbow Markers (*M*r 12 000-225 000) (GE Healthcare). Briefly, the run was conducted applying 60 mV for 30 min until protein samples completely entered in the running gel. The gels were then incubated for 1 h in fixing solution (10% (*v*/*v*) acetic acid, 50% (*v*/*v*) methanol), stained for 1 h with Coomassie Brilliant Blue (CBB) (0.1% (*w*/*v*) CBB R-250, 10% (*v*/*v*) acetic acid), and destained in 10% (*v*/*v*) acetic acid. 

The portion of gel containing proteins was excised and subjected to tryptic digestion. In-gel digestion was performed as previously described [20] with the following refinements. The volumes of solutions were adjusted to completely cover all gel samples (previously cut into 12 portions), and each sample was treated with 3 µg of trypsin (V5111, Promega, Madison, WI, USA). The extracted peptides were suspended in 0.1% (*v*/*v*) formic acid (FA). All of the procedures were replicated on three independent biological samples (*n* = 3).

### 3.4. Mass Spectrometry Analysis

All mass spectrometry experiments were conducted on an Agilent 6520 Q-TOF mass spectrometer equipped with an HPLC Chip Cube source driven by a 1200 series nano/capillary LC system (Agilent Technologies, Cernusco Sul Naviglio, MI, Italy)). Both systems were controlled by a MassHunter Workstation (version B.02.01, B2116.20; Agilent Technologies). Chromatography was performed into Polaris-HR-Chip-3C18 (Agilent Technologies), consisted of a 360-nL trap column and a 75 μm × 150-mm analytical column (Polaris C18-A, 180 Å, 3 µm). An aliquot of sample was loaded onto the trap column at 2 µL·min^−1^ in 0.1% (*v*/*v*) FA. The peptides were then eluted during a 100-min non-linear gradient of acetonitrile (from 3% to 50% *v*/*v*) in 0.1% (*v*/*v*) FA at 0.4 µL·min^−1^. The mass spectrometer ran in positive ion mode and MS scans were acquired over a range from 300 to 3000 mass-to-charge ratio (*m*/*z*) at 4 spectra·s^−1^. Precursor ions were selected by auto-MS/MS with a maximum of 4 precursors per cycle and active exclusion set at 2 spectra for 0.1 min. 

Analysis of MS/MS spectra were performed by Spectrum Mill MS Proteomics Workbench (Rev B.04.00.127; Agilent Technologies). Cysteine carbamidomethylation and methionine oxidation were used as fixed and variable modifications, admitting 2 tryptic missed cleavages per peptide. The search was conducted against the database of Zea mays (ID 4577) protein sequences (Aug 2017, 130162 entries) downloaded from UniProtKB/Swiss-Prot (http://www.uniprot.org/), and concatenated with the reverse one. The threshold used for protein identification was false discovery rate (FDR) ≤ 1%, number of unique peptides (NUP) ≥ 2, protein amino acid coverage ≥ 5% if NUP < 4. Peptide quantification was obtained as the spectrum intensity (SI) of the precursor (MH^+^). Protein quantification was obtained summing the SI of all the identified peptides in the protein. Protein abundance was normalized as the % with respect to the abundance of all validated proteins in the sample (%SI). Proteins showing at least a two-fold change in their %SI among at least 2 of all the experimental conditions were analysed according to the two-way ANOVA test to ascertain the source of variations and then by one-way ANOVA to assess the significance of the differences (Tukey post hoc, *p* < 0.05).

## Figures and Tables

**Table 1:** Total nitrogen (N) in plants and the ratio between the inorganic and organic forms in roots and leaves. Maize plants were collected at t0 and after 6 h, 30 h, and 54 h of growth in presence of 5 mM NO_3_^−^ (n), 5 mM NH_4_^+^ (a), and 2.5 mM NO_3_^−^ + 2.5 mM NH_4_^+^ (na). Values are the mean ± SE (*n* = 3). The statistical significance was assessed by ANOVA test (*p* < 0.05, Tukey post hoc method).

Condition	Time (h)	Total N Plant^−1^ (mg g^−1^ FW)	Inorganic N/Organic N Roots	Inorganic N/Organic N Leaves
	0	3.37 ± 0.02 (a)	0.50 ± 0.07 (bc)	0.24 ± 0.03 (abc)
Nitrate (n)	6	3.73 ± 0.06 (ab)	0.80 ± 0.01 (de)	0.23 ± 0.01 (ab)
	30	4.25 ± 0.10 (cd)	1.28 ± 0.10 (f)	0.26 ± 0.02 (bc)
	54	4.38 ± 0.10 (cd)	0.91 ± 0.07 (e)	0.34 ± 0.01 (c)
Ammonium (a)	6	4.13 ± 0.04 (bc)	0.49 ± 0.08 (bc)	0.28 ± 0.01 (bc)
	30	4.55 ± 0.03 (de)	0.34 ± 0.03 (ab)	0.21 ± 0.01 (ab)
	54	4.50 ± 0.06 (cde)	0.13 ± 0.01 (a)	0.14 ± 0.01 (a)
Nitrate + Ammonium (na)	6	4.34 ± 0.14 (cd)	0.74 ± 0.02 (cde)	0.28 ± 0.03 (bc)
	30	4.90 ± 0.10 (e)	0.74 ± 0.07 (cde)	0.28 ± 0.02 (bc)
	54	4.60 ± 0.06 (de)	0.61 ± 0.04 (bcd)	0.30 ± 0.04 (bc)

**Table 2:** Evaluation of the comparative proteomic analyses in roots and leaves of maize.

Parameter	Root	Leaf
*n*. of total peptides	39,516	31,739
Average of peptides per sample (±SE, *n* = 27)	1464 ± 15	1176 ± 11
Average of peptides per condition (±SE, *n* = 9)	4391 ± 55	3527 ± 50
Average of unique peptide per protein (±SE)	6.8 ± 0.3	6.4 ± 0.4
Average amino acid coverage % (±SE)	22.5 ± 0.8	22.4 ± 0.9
*n*. of identified proteins	336	246
*n*. of differentially accumulated proteins (%)	51 (15%)	48 (19%)

**Table 3:** Proteins differentially accumulated in root proteome. Proteins are grouped according to the functional classifications. FC: maximum fold change among conditions, n.d.: not detectable, the protein was absent in at least one condition. V: main source of variation (two-way ANOVA, *p* < 0.05); t: time, s: N source; i: interaction. Differences: h: hours of exposure to 5 mM NO_3_^−^ (n), 5 mM NH_4_^+^ (a), and 2.5 mM NO_3_^−^ + 2.5 mM NH_4_^+^ (na). ^a^: annotated by basic local alignment search tool (BLAST). Different letters indicate significant difference (* *p* < 0.05, Tukey post-hoc); letters are arranged in ascending order according to the increase in protein abundance. Bold letters indicate significant difference within each N source. NADH: Nicotinamide adenine dinucleotide; NADP: Nicotinamide adenine dinucleotide phosphate; PIP: plasma membrane intrinsic proteins; TIP: tonoplast intrinsic proteins.

ID	Entry—Protein Name	FC				Differences								
			V			h (n)			h (a)			h (na)		
			t	s	i	06	30	54	06	30	54	06	30	54
Nitrogen assimilation														
R74	**B6SY01**—Ferredoxin-nitrite reductase	**7.57**			*	**abc**	**d**	**cd**	abc	ab	a	abc	bcd	abc
R64	**B4FR61**—Glutamine synthetase (cytosol)	**2.60**			*	a	a	a	**ab**	**c**	**c**	**a**	**a**	**cb**
R189	**A0A1D6NFK0**—Glutamate synthase 1 [NADH] chloroplastic	**8.54**	*	*		a	ab	a	**a**	**ab**	**b**	a	a	ab
R103	**B5U8J8**—Asparagine synthetase	**n.d.**			*	a	a	a	**a**	**b**	**c**	a	a	a
R68	**B4G043**—Ferredoxin-NADP reductase	**5.48**			*	cb	cb	c	**cb**	**ab**	**a**	cb	ab	abc
**Amino acid metabolism**														
R6	**B6U4D6**—Phenylalanine ammonia-lyase	**2.88**			*	bc	abc	bc	**bc**	**a**	**a**	**c**	**abc**	**ab**
R107	**P80608**—Cysteine synthase	**2.74**			*	**a**	**bc**	**ab**	**ab**	**ab**	**c**	**a**	**bc**	**abc**
R49	**A0A1D6FUX8**—*S*-adenosylmethionine synthase	**2.16**	*			**b**	**a**	**ab**	ab	ab	ab	b	ab	ab
R96	**K7TSD2**—Serine hydroxymethyltransferase	**2.08**	*			b	ab	ab	ab	ab	a	ab	a	a
**Carbon metabolism**														
R207	**C0PMR3**—Glucose-6-phosphate 1-dehydrogenase (cytosol)	**7.66**	*			ab	b	ab	a	ab	ab	ab	ab	ab
R85	**A0A1D6J424**—Glucose-6-phosphate 1-dehydrogenase (chloroplast)	**224**		*		ab	b	ab	ab	a	a	ab	ab	ab
R57	**A0A1D6NVZ7**—Transketolase 1	**2.98**	*	*		ab	a	ab	ab	a	a	**b**	**ab**	**a**
R231	**Q8L5G8**—Phosphotransferase	**7.59**	*			ab	b	ab	a	ab	ab	a	ab	ab
R168	**A0A1D6NR86**—Pyrophosphate-fructose 6-phosphate 1-phosphotransferase subunit beta	**2.85**			*	**a**	**b**	**ab**	a	a	a	**a**	**b**	**ab**
R243	**B4F820**—Triosephosphate isomerase **^a^**	**3.67**	*			a	ab	abc	**a**	**ab**	**c**	abc	abc	bc
R19	**C0HHU2**—2,3-bisphosphoglycerate-independent phosphoglycerate mutase 1	**2.35**			*	a	ab	ab	**a**	**ab**	**c**	**a**	**a**	**cb**
R119	**A0A1D6H4C4**—Citrate synthase	**2.87**			*	ab	ab	a	**a**	**ab**	**b**	a	ab	a
R28	**Q9SAZ6**—Phosphoenolpyruvate carboxylase	**3.20**	*	*		a	ab	a	ab	ab	b	a	ab	ab
**Cell water homeostasis**														
R290	**B6T7A1**—Voltage-gated potassium channel beta subunit	**2.21**	*	*		**ab**	**c**	**bc**	ab	ab	ab	a	abc	abc
R127	**B6T634**—Aquaporin PIP2.1	**2.75**			*	ab	ab	b	ab	ab	a	ab	b	b
R193	**A0A1R3N4Y1**—Aquaporin PIP2-5	**5.52**	*	*		**a**	**abc**	**c**	ab	abc	ab	abc	abc	bc
R285	**B6TNY0**—Aquaporin TIP2.1	**2.81**	*	*		ab	ab	b	ab	a	ab	ab	ab	ab
**Cell wall metabolism**														
R92	**A0A1D6IMH7**—Hydroxyproline-rich glycoprotein family protein	**4.07**			*	**b**	**a**	**a**	ab	ab	ab	ab	a	ab
R154	**B6UD26**—*O*-methyltransferase ZRP4	**3.66**			*	ab	ab	ab	**a**	**a**	**b**	ab	ab	ab
**Protein synthesis and folding**														
R58	**C0PCQ6**—40S ribosomal protein S2-1	**3.21**	*			**c**	**a**	**ab**	bc	abc	abc	bc	ab	abc
R255	**A0A1D6PYT7**—Ribosomal protein S25	**2.80**			*	bc	ab	ab	**c**	**abc**	**a**	abc	ab	ab
R309	**A0A1D6FKZ4**—60S acidic ribosomal protein P2-5	**6.66**	*			**a**	**b**	**ab**	ab	ab	a	ab	ab	ab
R87	**C4JA45**—60S ribosomal protein L5-1 homolog b	**2.52**	*			**a**	**b**	**b**	ab	ab	b	ab	b	ab
R122	**B6T267**—Ribosomal protein L15	**18.7**	*			bc	ab	abc	c	abc	abc	abc	a	abc
R4	**B7ZZ42**—Heat shock 70 kDa protein 3	**2.25**			*	**bc**	**a**	**abc**	bc	abc	ab	**c**	**a**	**ab**
R100	**B6U0V6**—Endoplasmin	**5.19**	*			ab	ab	ab	a	ab	a	ab	b	ab
R188	**B6SXW8**—RuBisCO large subunit-binding protein sub. alpha	**3.92**	*			a	ab	ab	a	ab	a	**a**	**b**	**ab**
R297	**A0A1D6FAH0**—T-complex protein 1 subunit epsilon	**4.66**	*			ab	ab	a	ab	ab	a	ab	b	ab
R131	**B4FZZ2**—Peptidyl-prolyl cis-trans isomerase	**4.68**	*			**c**	**a**	**ab**	**c**	**a**	**ab**	**bc**	**a**	**ab**
R216	**A0A1D6LN79**—Peptidyl-prolyl cis-trans isomerase	**15.7**	*			**d**	**a**	**abc**	cd	abcd	abcd	bcd	ab	abcd
R145	**A0A1D6F8L7**—Coatomer subunit gamma	**3.43**	*			a	ab	ab	a	a	a	**a**	**b**	**a**
R205	**A0A1D6PJW1**—Proteasome subunit alpha type	**n.d.**	*	*		a	a	a	**b**	**a**	**a**	a	a	a
R230	**B4FB55**—Ras-related protein ARA-3	**2.40**	*	*		ab	b	b	a	ab	ab	ab	b	ab
**Stress response and other functions**														
R105	**C0P848**—Formate dehydrogenase, mitochondrial	**14.2**	*			a	a	a	**a**	**a**	**b**	a	ab	ab
R99	**B8A2B4**—Guanine nucleotide-binding protein beta subunit-like protein **^a^**	**2.22**	*			**abc**	**a**	**c**	**ab**	**a**	**bc**	bc	ab	bc
R112	**A5H452**—Peroxidase 70	**2.75**	*			**c**	**a**	**abc**	abc	abc	abc	bc	ab	ab
R133	**A0A1D6E530**—Peroxidase	**3.56**	*			ab	b	ab	ab	ab	ab	a	ab	ab
R300	**B6SIU4**—Peroxidase	**n.d.**			*	**bc**	**ab**	**c**	abc	ab	a	abc	abc	ab
R31	**C0PH85**—Tubulin beta chain	**2.88**	*	*		**d**	**cb**	**cb**	**c**	**ab**	**a**	c	bc	abc
R79	**Q6RW10**—Allene oxide synthase	**2.27**			*	**a**	**b**	**ab**	a	ab	ab	ab	ab	ab
R225	**A0A1D6HSR3**—Putative carboxylesterase 15	**n.d.**			*	a	a	a	**a**	**b**	**c**	**a**	**ab**	**b**
R236	**A0A1D6QNT6**—Peroxisomal fatty acid beta-oxidation multifunctional protein **^a^**	**3.86**	*			a	ab	ab	ab	ab	ab	**a**	**b**	**ab**
R271	**Q71RX2**—Isopentenyl pyrophosphate isomerase	**n.d.**	*	*		**ab**	**c**	**bc**	a	abc	abc	**a**	**bc**	**bc**
R272	**B6TKK2**—Gibberellin receptor GID1L2	**n.d.**	*			ab	ab	b	ab	ab	ab	a	a	ab
R301	**B6TP36**—Prohibitin	**3.56**	*			ab	bc	abc	a	abc	abc	**ab**	**c**	**abc**
R318	**B6T7H0**—Glutathione S-transferase 6	**n.d.**			*	**a**	**ab**	**b**	a	a	a	a	a	a

**Table 4:** Proteins differentially accumulated in leaf proteome. Proteins are grouped according to the functional classifications. FC: maximum fold change among conditions, n.d.: not detectable, the protein was absent in at least one condition. V: main source of variation (two-way ANOVA, *p* < 0.05); t: time, s: N source; i: interaction. Differences: h: hours of exposure to 5 mM NO_3_^−^ (n), 5 mM NH_4_^+^ (a), and 2.5 mM NO_3_^−^ + 2.5 mM NH_4_^+^ (na). ^a^: annotated by BLAST. Different letters indicate significant difference (* *p* < 0.05, Tukey post-hoc); letters are arranged in ascending order according to the increase in protein abundance. Bold letters indicate significant difference within each N source. ATP: adenosine triphosphate; HSP: heat shock protein.

ID	Entry—Protein Name	FC				Differences								
			V			h (n)			h (a)			h (na)		
			t	s	i	06	30	54	06	30	54	06	30	54
Nitrogen assimilation														
L149	**B6SY01**—Ferredoxin-nitrite reductase	**3.00**	*			**a**	**ab**	**b**	a	a	ab	ab	a	ab
L177	**B6TE43**—Glutamine synthetase (chloroplast)	**3.92**	*			ab	ab	ab	ab	a	a	**b**	**ab**	**a**
**Amino acid metabolism**														
L193	**B6TQ06**—Aminomethyltransferase	**2.31**	*			ab	ab	b	ab	a	ab	ab	ab	ab
L22	**C0P5Y3**—5-methyltetrahydropteroyltriglutamate -homocysteine methyltransferase 1 (MetE)	**3.57**	*			ab	a	ab	ab	ab	b	ab	ab	ab
L194	**A0A1D6KDZ0**—Shikimate kinase family protein	**2.85**	*			**abc**	**ab**	**c**	**ab**	**ab**	**c**	**abc**	**a**	**bc**
**Photosynthesis**														
L31	**P04966**—Photosystem I P700 chlorophyll a apoprotein A1	**2.48**	*			**a**	**ab**	**b**	**a**	**b**	**ab**	ab	b	ab
L85	**P04967**—Photosystem I P700 chlorophyll a apoprotein A2	**2.62**			*	**a**	**bc**	**ab**	**ab**	**c**	**ab**	ab	ab	ab
L132	**B6U534**—Photosystem I reaction center subunit V	**3.85**	*			**c**	**c**	**a**	**bc**	**c**	**a**	bc	bc	ab
L196	**B4FLT7**—Photosystem I H subunit1	**2.99**	*	*		**bcd**	**d**	**ab**	**abc**	**cd**	**a**	**ab**	**bcd**	**a**
L113	**B6SP64**—Oxygen evolving enhancer protein 3 containing protein	**2.34**	*			**a**	**a**	**b**	**a**	**a**	**b**	**a**	**a**	**b**
L162	**B8A3D1**—Pyruvate phosphate dikinase regulatory protein, chloroplastic **^a^**	**6.68**	*			a	a	ab	**a**	**a**	**b**	a	a	ab
L212	**B6TKB3**—Triose phosphate/phosphate translocator, non-green plastid, chloroplast	**n.d.**	*			a	a	ab	**a**	**a**	**b**	**a**	**a**	**b**
**Energy metabolism**														
L115	**B6T908**—ATP synthase B chain	**8.19**	*	*		**ab**	**abc**	**c**	a	ab	abc	ab	abc	bc
L225	**B6SP77**—ATP synthase C chain	**77.8**	*			**a**	**a**	**b**	a	a	ab	a	a	ab
L155	**B6T168**—ATP synthase epsilon chain	**3.09**	*			**a**	**abc**	**bc**	**a**	**abc**	**bc**	**abc**	**ab**	**c**
L130	**B4FK49**—Nucleoside diphosphate kinase	**4.88**	*	*		a	ab	abc	abc	abc	bc	**abc**	**ab**	**c**
L236	**C0HHC4**—Nucleoside diphosphate kinase	**7.24**	*			**a**	**a**	**b**	**a**	**a**	**b**	**a**	**a**	**b**
**Carbon metabolism**														
L192	**A0A1D6N8I0**—2,3-bisphosphoglycerate-independent phosphoglycerate mutase 1	**4.77**	*			ab	a	ab	ab	ab	ab	ab	ab	b
L191	**B8A0W7**—Enolase 1 **^a^**	**13.1**	*			a	a	abc	**ab**	**a**	**bc**	**a**	**a**	**c**
L110	**B4FZU8**—Malate dehydrogenase	**2.31**	*			**a**	**a**	**b**	**a**	**a**	**b**	**a**	**a**	**b**
**Protein synthesis and folding**														
L69	**A0A0B4J303**—Eukaryotic initiation factor4a	**3.01**	*			abc	a	abc	ab	ab	bc	**ab**	**a**	**c**
L111	**A0A1D6LAB8**—RNA helicase4	**3.98**			*	a	a	a	a	a	a	**a**	**a**	**b**
L205	**A0A1X7YHC0**—30S ribosomal protein S2, chloroplastic **^a^**	**n.d.**	*			ab	ab	a	ab	ab	a	**b**	**ab**	**a**
L127	**C0PEC4**—30S ribosomal protein S5 chloroplastic	**4.70**	*			ab	ab	b	**ab**	**a**	**b**	ab	ab	ab
L211	**P08530**—30S ribosomal protein S8, chloroplastic	**3.27**	*			**ab**	**a**	**bc**	ab	ab	bc	**ab**	**a**	**c**
L137	**B4FR40**—30S ribosomal protein S9 chloroplastic	**n.d.**	*			ab	a	a	**ab**	**b**	**a**	ab	ab	a
L145	**B6UGL6**—40S ribosomal protein S5	**2.27**	*	*		**a**	**a**	**b**	**a**	**a**	**b**	**a**	**a**	**b**
L118	**A0A1D6P3R8**—40S ribosomal protein S14	**2.15**			*	ab	a	ab	ab	b	ab	a	ab	ab
L88	**B6SNQ7**—40S ribosomal protein S16	**n.d.**			*	a	a	ab	**a**	**a**	**b**	ab	a	ab
L171	**B6UF84**—50S ribosomal protein L3	**2.23**	*			ab	a	ab	ab	ab	ab	ab	ab	b
L195	**B6SK79**—60S ribosomal protein L4	**3.28**	*			ab	a	ab	ab	ab	ab	ab	b	b
L216	**B6SHW0**—60S ribosomal protein L6	**2.33**	*			a	a	ab	ab	a	ab	**a**	**ab**	**b**
L126	**A0A1D6GM13**—60S ribosomal protein L10 **^a^**	**2.40**	*			ab	a	ab	ab	ab	ab	**ab**	**a**	**b**
L239	**B6TM00**—60 ribosomal protein L14	**2.66**	*	*		**ab**	**a**	**bc**	bc	bc	bc	**ab**	**ab**	**c**
L240	**B6SJH2**—60S ribosomal protein L17	**2.65**	*			**a**	**a**	**b**	**a**	**a**	**b**	**a**	**a**	**b**
L15	**A0A1X7YIM9**—RuBisCO large subunit-binding protein subunit alpha, chloroplastic **^a^**	**2.10**	*			**c**	**a**	**bc**	c	abc	c	**c**	**ab**	**c**
L19	**B7ZZ42**—Heat shock 70 kDa protein 3	**2.73**		*		ab	a	ab	ab	b	ab	b	ab	b
L64	**C3UZ63**—HSP protein	**6.72**	*			**abc**	**a**	**cb**	abc	abc	bc	**ab**	**abc**	**c**
L70	**A0A1D6LIK1**—Peptidyl-prolyl cis-trans isomerase	**2.04**	*			**d**	**abcd**	**a**	**cd**	**abc**	**ab**	**bcd**	**abcd**	**a**
L204	**B4FZZ2**—Peptidyl-prolyl cis-trans isomerase	**n.d.**	*			**b**	**a**	**a**	**c**	**a**	**a**	**bc**	**a**	**a**
L243	**A0A1D6FAW5**—Peptidyl-prolyl cis-trans isomerase	**n.d.**	*			**b**	**a**	**a**	**b**	**a**	**a**	**b**	**a**	**a**
L154	**B6TCN7**—Threonine endopeptidase	**3.58**			*	a	ab	abc	**a**	**a**	**c**	**ab**	**a**	**bc**
**Stress response and other functions**														
L76	**A1XCI5**—Lipoxygenase	**4.44**	*	*		a	a	a	**a**	**a**	**b**	a	a	ab
L189	**B6T2W9**—Thylakoid lumenal 19 kDa protein	**2.84**			*	**b**	**a**	**ab**	a	ab	ab	ab	a	ab
L201	**B6SU36**—Thylakoid lumenal 16.5 kDa protein	**10.3**			*	**a**	**ab**	**b**	ab	a	ab	ab	ab	ab
L143	**B6T7W8**—Cortical cell-delineating protein	**2.57**	*			**ab**	**a**	**b**	ab	ab	b	**ab**	**a**	**b**
L180	**K7UGI3**—Putative thioredoxin superfamily protein	**4.52**	*			**abc**	**a**	**bcd**	**abcd**	**ab**	**cd**	**ab**	**a**	**d**
L208	**C4J9Y2**—Aluminum-induced protein homolog1	**10.5**	*			**ab**	**a**	**b**	a	a	ab	ab	a	ab

## Supplementary Materials

Supplementary materials can be found at http://www.mdpi.com/1422-0067/19/8/2202/s1. Supplementary Data 1. Comparative proteomic profiles of maize plants. The file reports data about the comparative characterization of the root (Supplementary Table S1) and leaf (Supplementary Table S2) proteomes. Supplementary Data 2. Changes in the level of proteins differentially accumulated in roots and leaves of maize plants. The file reports as bar charts the levels of the proteins differentially accumulated in roots and leaves of maize plants during the different nutritional treatments.

## Abbreviations

2-ME	2-Mercaptoethanol
CBB	Coomassie Brilliant Blue
DPs	Differentially abundant Proteins
FA	Formic acid
NUP	Number of unique peptides
OPA	*o*-Phthalaldehyde
SDS	Sodium dodecyl sulfate
SI	Spectrum intensity
