# Supplementary material for: Time-Course of Metabolic and Proteomic Responses to Different Nitrate/Ammonium Availabilities in Roots and Leaves of Maize
Source: Int J Mol Sci. 2018 Jul 27;19(8):2202. doi: 10.3390/ijms19082202 (PMC6121299; doi:10.3390/ijms19082202)

Supplementary Data 2.

Changes in the level of proteins differentially accumulated in roots and leaves of maize plants. The file reports as bar charts the levels of the proteins differentially accumulated in roots and leaves of maize plants during the different nutritional treatments. The proteins were grouped according to functional classification (Figure 4, Tables 2 and 3). Plants were exposed for 6 h (light grey bars), 30 h (grey bars) and 54 h (dark grey bars) to 5 mM NO_3^- (nitrate), 5 mM NH_4^+ (ammonium) and 2.5 mM NO_3^- + 2.5 mM NH_4^+ (nitrate/ammonium). %SI: % of the protein spectrum intensity respect the sum of all validated proteins in the sample. Values are the mean ± SE (n=3). The significance was assessed by ANOVA test ($p < 0.05$, *Tukey post-hoc*).

Nitrogen assimilation

Root

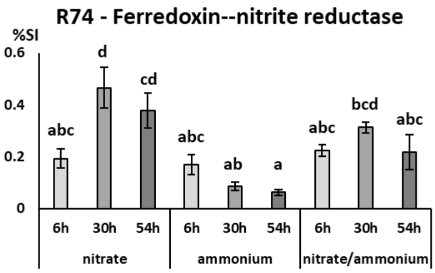

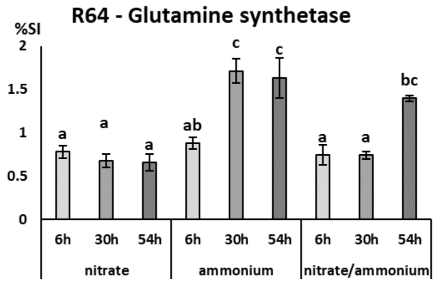

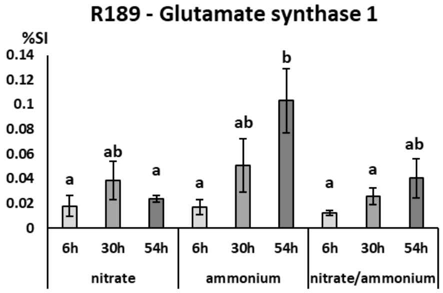

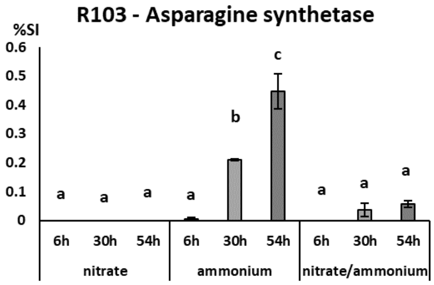

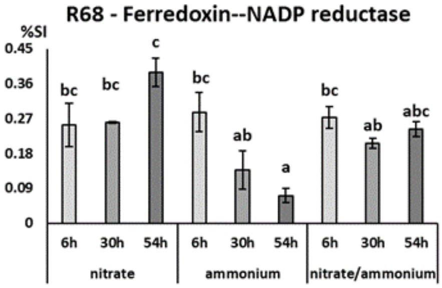

Leaf

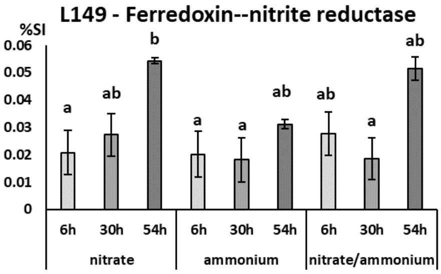

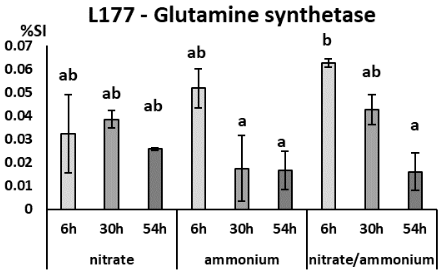

Amino acid metabolism

Root

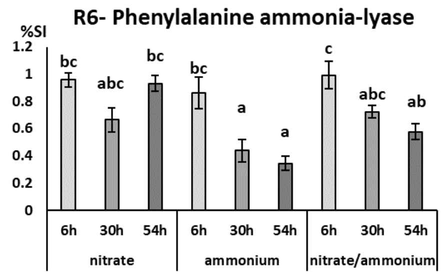

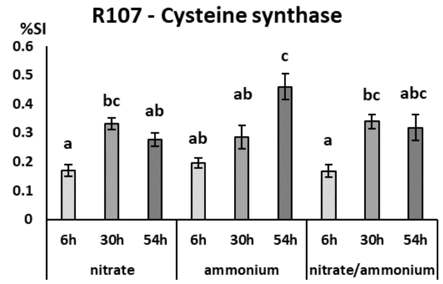

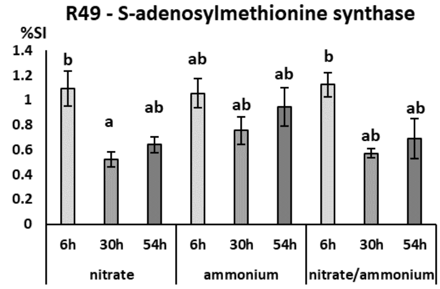

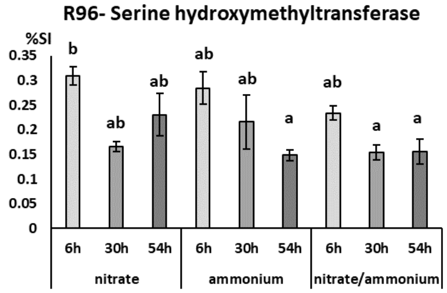

Leaf

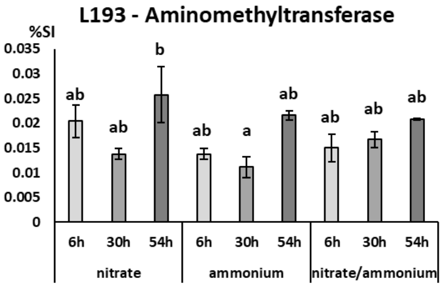

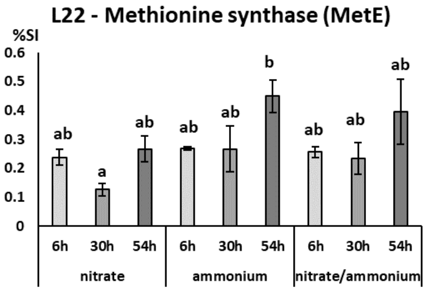

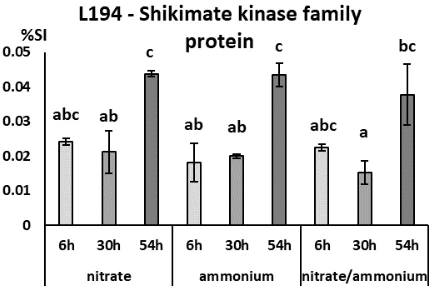

Photosynthesis: leaf

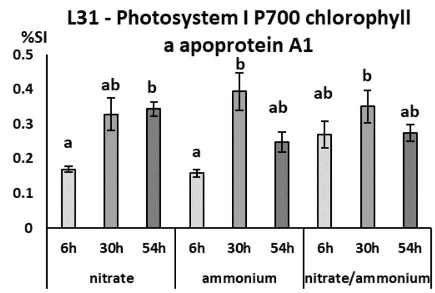

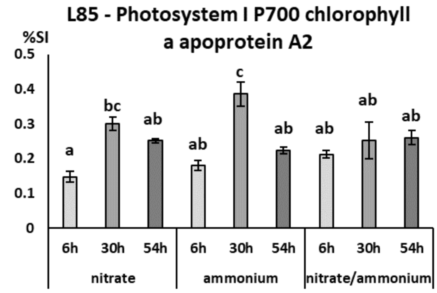

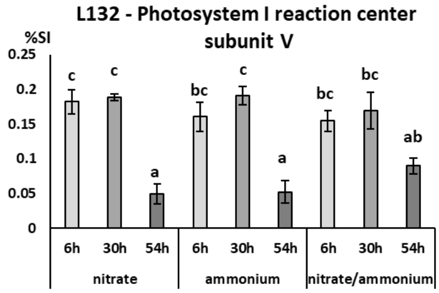

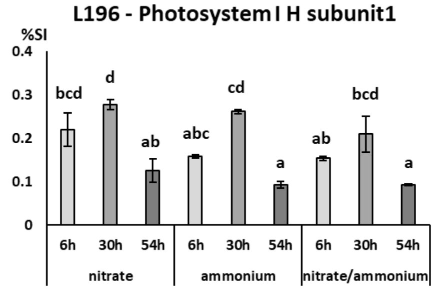

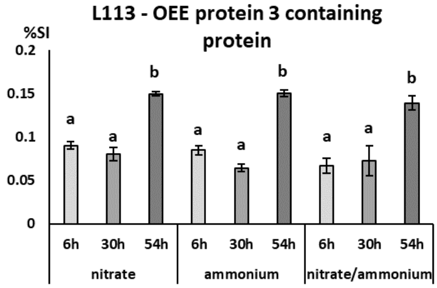

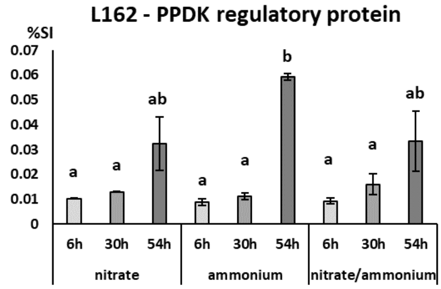

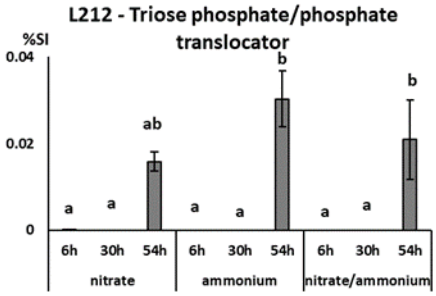

Energy metabolism

Leaf

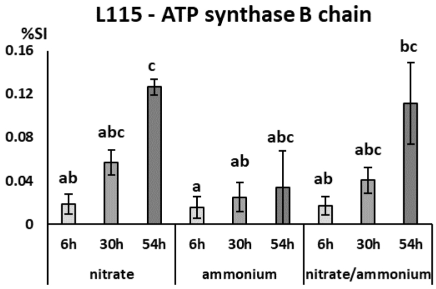

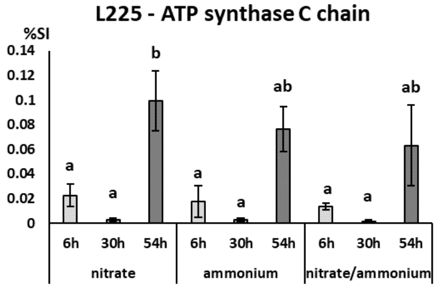

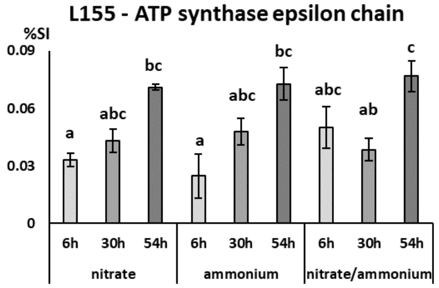

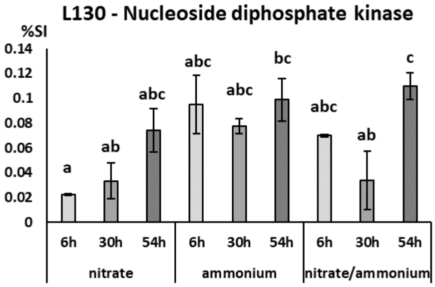

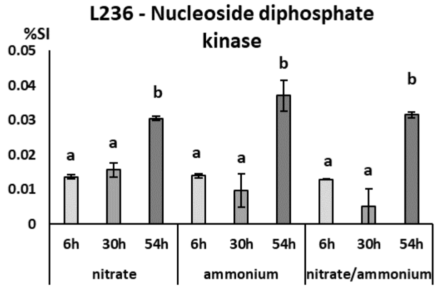

Carbon metabolism

Root

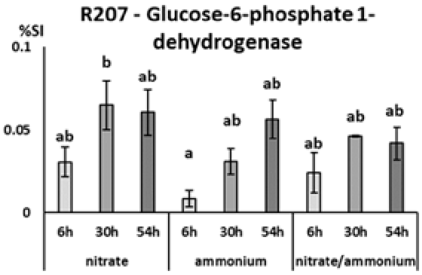

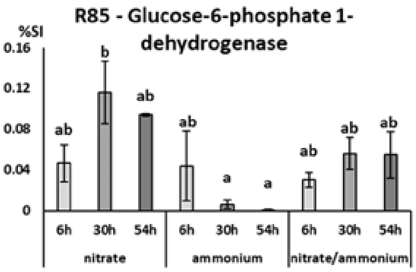

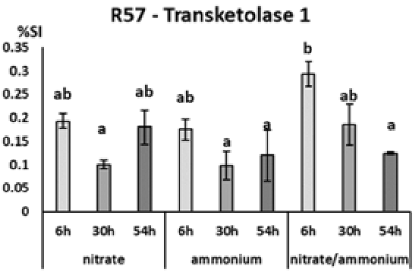

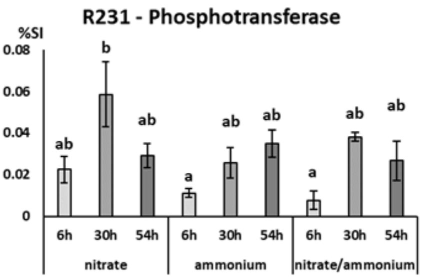

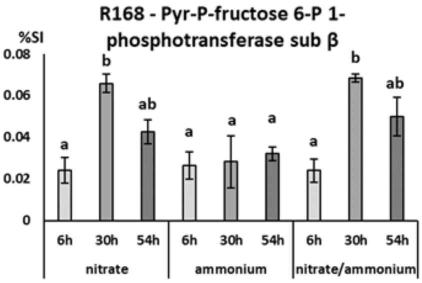

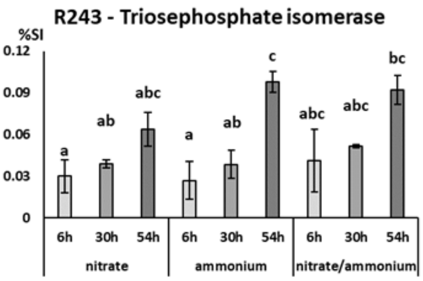

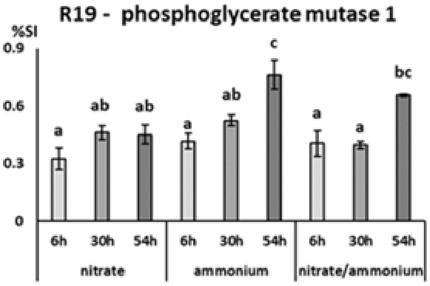

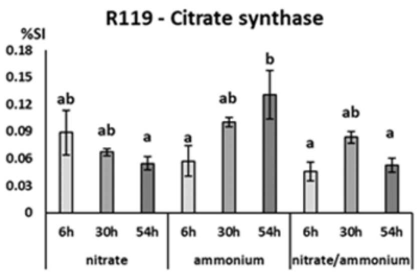

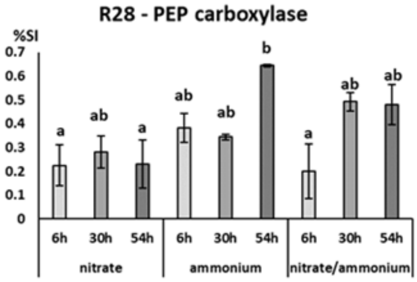

Leaf

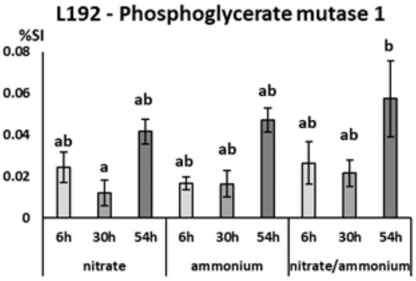

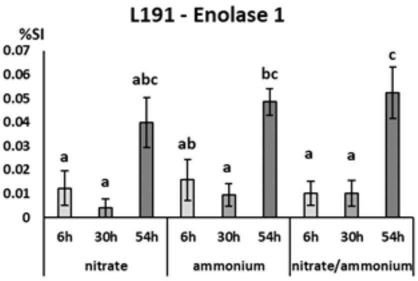

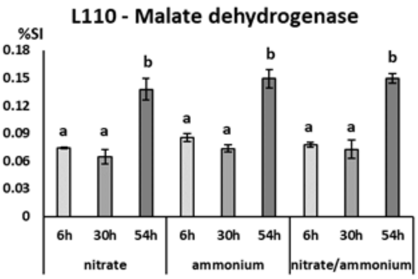

Cell water homeostasis: root

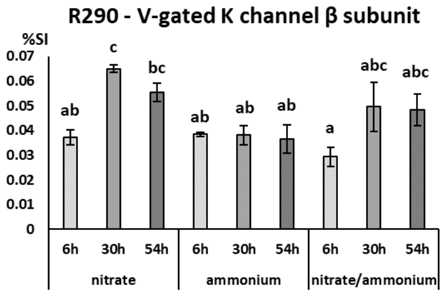

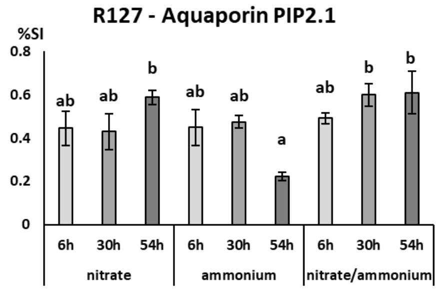

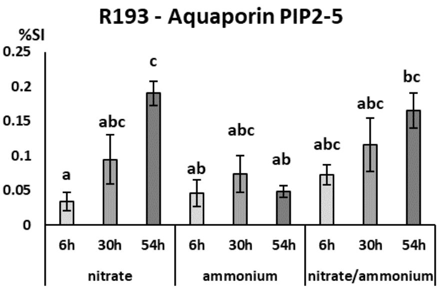

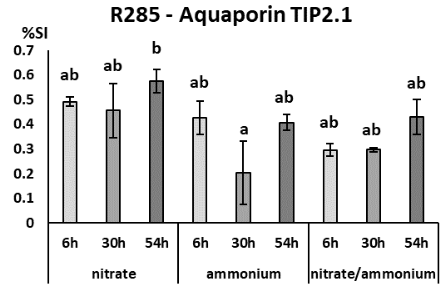

Cell wall metabolism: root

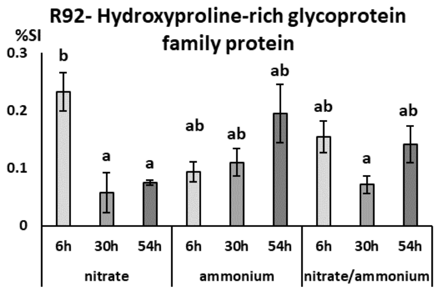

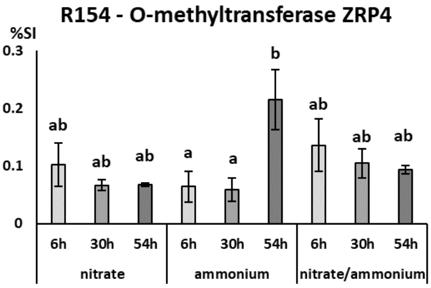

Protein synthesis and folding: root

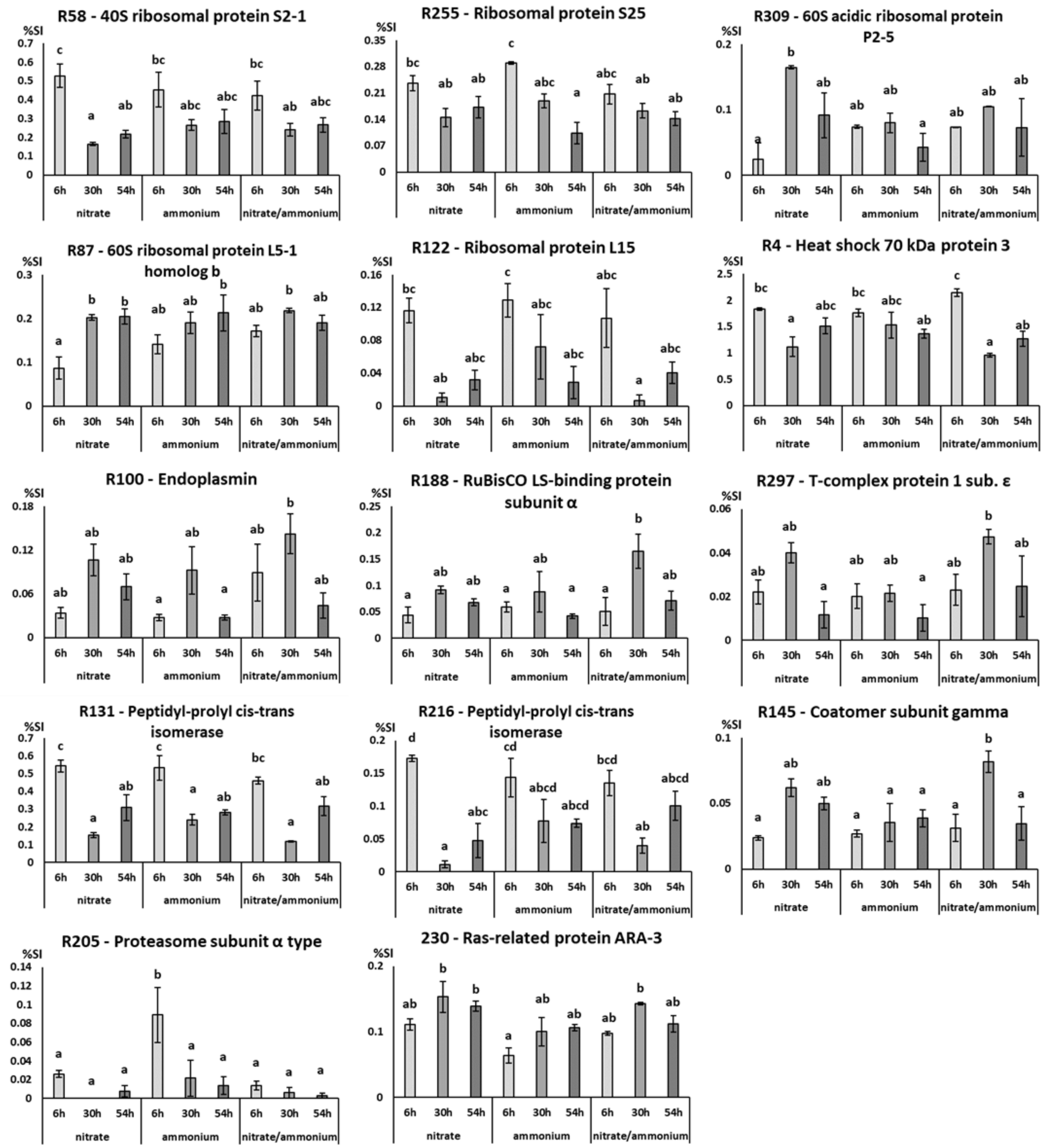

Protein synthesis and folding: leaf (1)

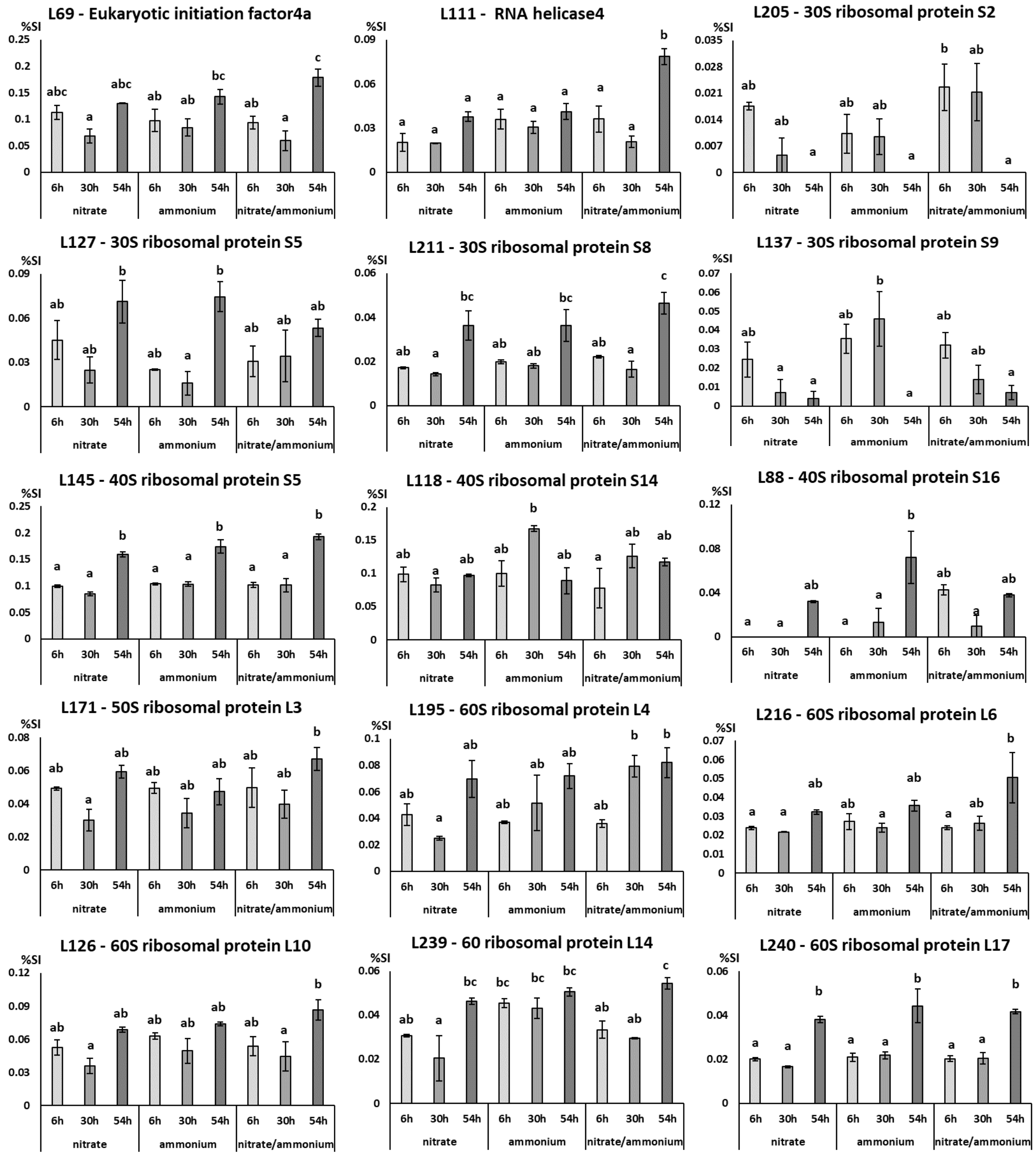

Protein synthesis and folding: leaf (2)

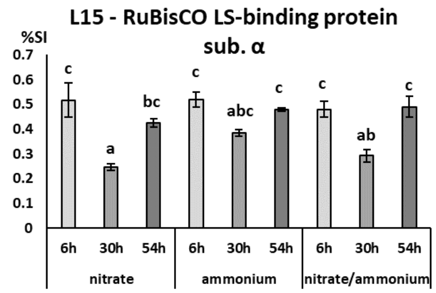

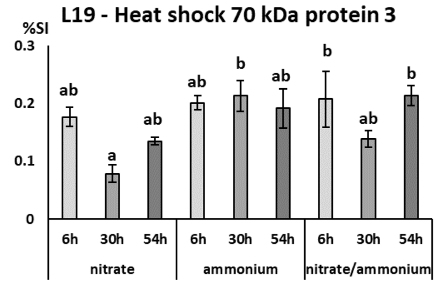

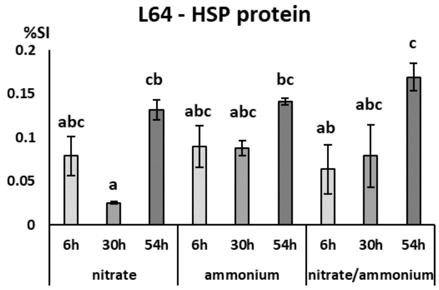

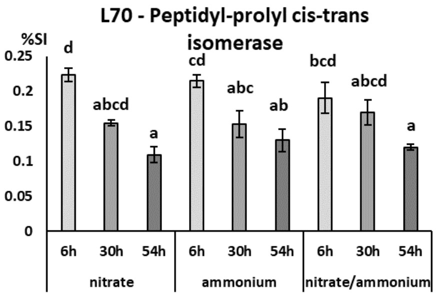

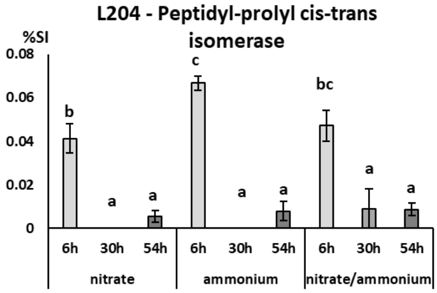

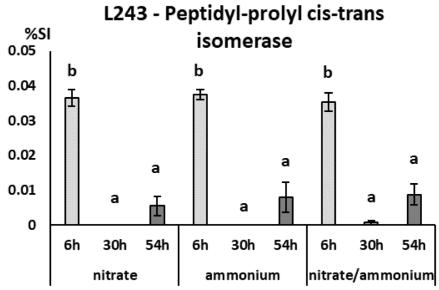

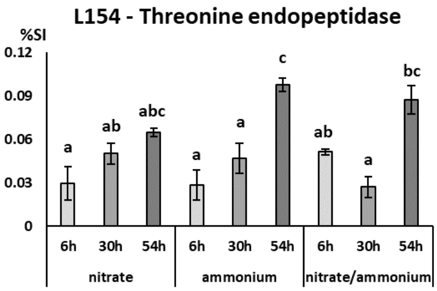

Stress response and other functions: root

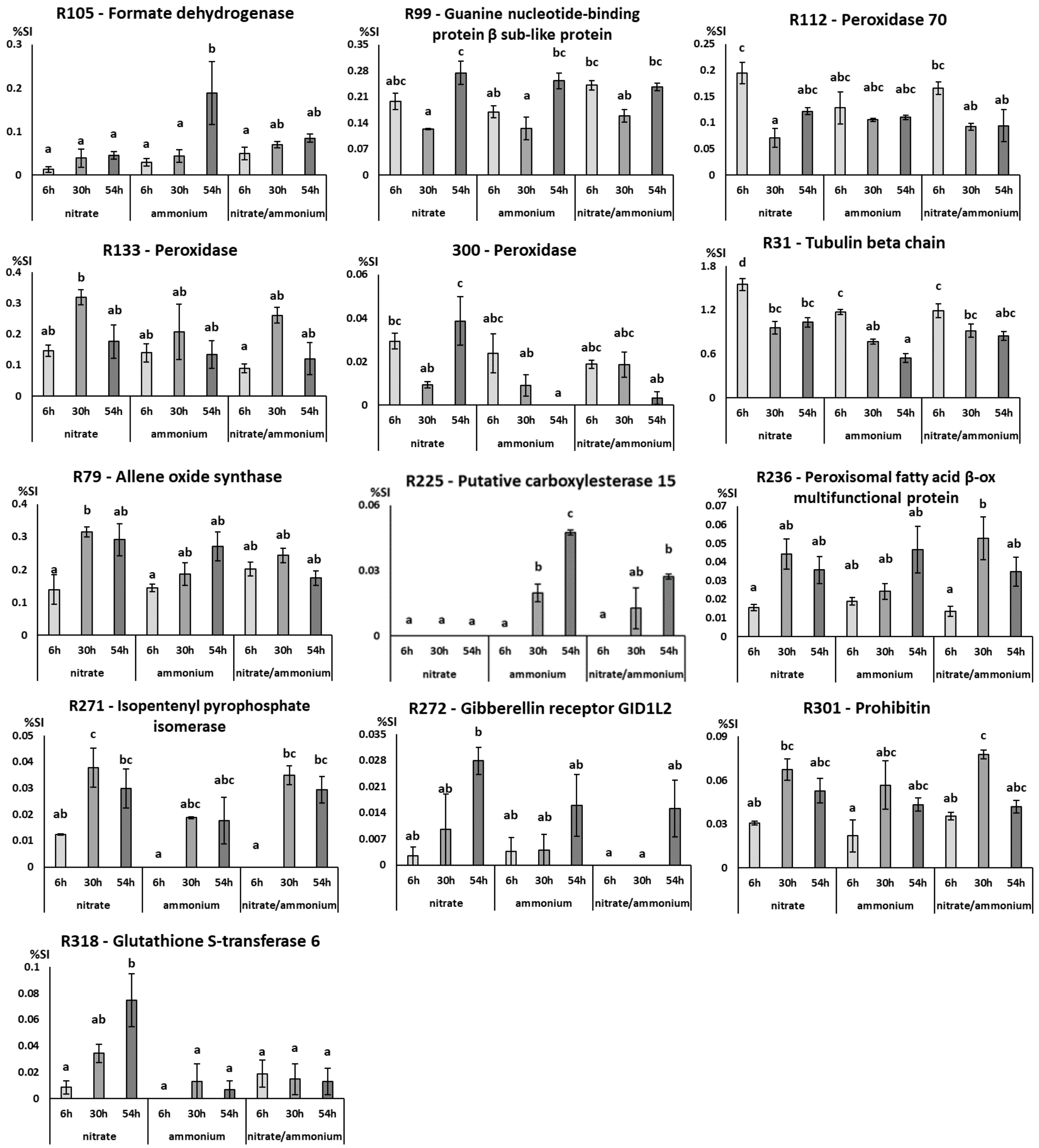

Stress response and other functions: leaf

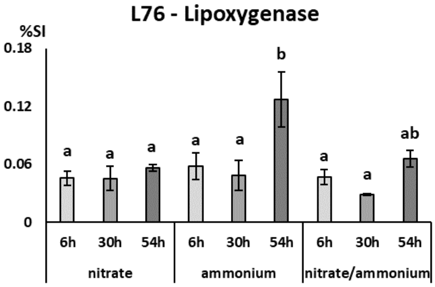

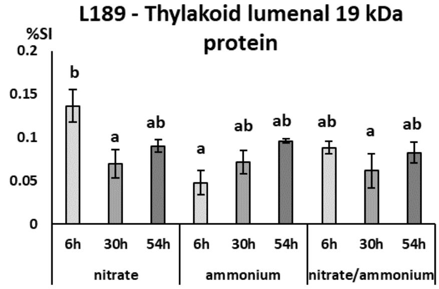

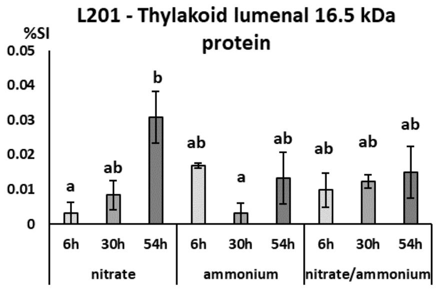

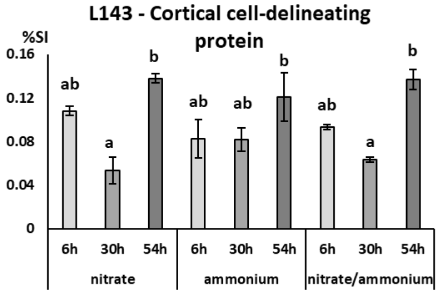

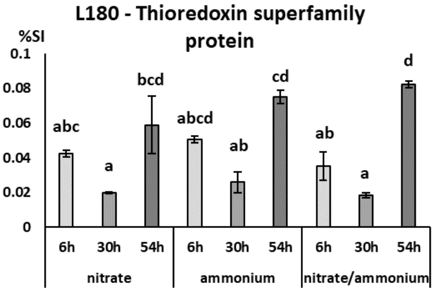

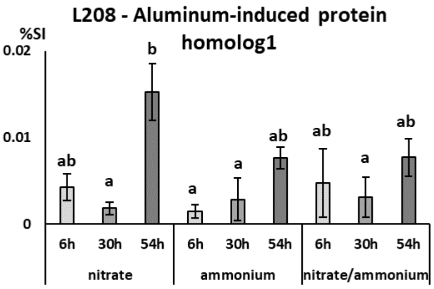

Supplement: Supplementary file 1 [file ijms-19-02202-s001.zip › Supplementary Data 2.pdf]
